# Cucurbitacins as potential anticancer agents: new insights on molecular mechanisms

**DOI:** 10.1186/s12967-022-03828-3

**Published:** 2022-12-31

**Authors:** Carla Varela, Catarina Melim, Beatriz G. Neves, Javad Sharifi-Rad, Daniela Calina, Assem Mamurova, Célia Cabral

**Affiliations:** 1 Chemical Process Engineering and Forest Products (CIEPQPF), Faculty of Medicine, University of Coimbra, Coimbra, Portugal; 2 Coimbra Institute for Clinical and Biomedical Research (iCBR), Clinic Academic Center of Coimbra (CACC), Faculty of Medicine, University of Coimbra, Coimbra, Portugal; 3 Center for Innovative Biomedicine and Biotechnology (CIBB), University of Coimbra, Coimbra, Portugal; 4 Facultad de Medicina, Universidad del Azuay, Cuenca, Ecuador; 5 Department of Clinical Pharmacy, University of Medicine and Pharmacy of Craiova, 200349 Craiova, Romania; 6 Department of Biodiversity of Bioresources, Al-Farabi Kazakh National University, Almaty, Kazakhstan; 7 Centre for Functional Ecology, Department of Life Sciences, University of Coimbra, Coimbra, Portugal

**Keywords:** Cucurbitacins, Chemical synthesis, Malignant tumor, Chemoprevention, Anticancer mechanisms, Molecular targets, Apoptosis

## Abstract

Since ancient times, plants have been an extensive reservoir of bioactive compounds with therapeutic interest for new drug development and clinical application. Cucurbitacins are a compelling example of these drug leads, primarily present in the plant kingdom, especially in the Cucurbitaceae family. However, these natural compounds are also known in several genera within other plant families. Beyond the Cucurbitaceae family, they are also present in other plant families, as well as in some fungi and one shell-less marine mollusc. Despite the natural abundance of cucurbitacins in different natural species, their obtaining and isolation is limited, as a result, an increase in their chemical synthesis has been developed by researchers. Data on cucurbitacins and their anticancer activities were collected from databases such as PubMed/MedLine, TRIP database, Web of Science, Google Scholar, and ScienceDirect and the information was arranged sequentially for a better understanding of the antitumor potential. The results of the studies showed that cucurbitacins have significant biological activities, such as anti-inflammatory, antioxidant, antimalarial, antimicrobial, hepatoprotective and antitumor potential. In conclusion, there are several studies, both in vitro and in vivo reporting this important anticancer/chemopreventive potential; hence a comprehensive review on this topic is recommended for future clinical research.

**Supplementary Information:**

The online version contains supplementary material available at 10.1186/s12967-022-03828-3.

## Introduction

Cancer is a worldwide public health problem that develops following an abnormal growth of potentially invasive cells, capable of infinite replication and metastasis [1–6]. It maintains a poor prognosis, with the yearly incidence and death rate steadily increasing over time, discovering new therapeutic molecules and strategies an ever-present necessity [7–12]. Opportunely, several biologically active natural molecules have been researched and applied for cancer treatment, including cucurbitacins and their derivatives which, are mainly organized in the groups: A, B, C, D, E, I, H, Q, R, and dihydrocucurbitacin B [13]. From the chemical point of view, cucurbitacins are cucurbitane-type tetracyclic triterpenoid saponins with 30 carbons atoms on their basic skeleton (the detailed chemical data can be found in Figures S1, S2 and S3 and Table 1, and the synthesis of cucurbitacins in Figures S4 to S12, in the Additional file 1).

Cucurbitacin B isolated from *Trichosanthes cucumerina* (snake gourd) is the greatest source of cucurbitacins, which ensued the attention of scientists because of its several anticancer mechanisms [14, 15]. There are numerous health benefits displayed by the entire Cucurbitaceae family, each vegetable or fruit with a unique effect on human health [16]. For example, the well-known vegetable *Cucumis sativus* (cucumber) rich in cucurbitacins A, B, C, D, and E, has been reported to alleviate symptoms of indigestion and constipation, to help with skin problems and to promote hair growth [17]. Cucurbits have been also to stimulate the central nervous system and to treat dizziness. Furthermore, their seeds have also been used in the treatment of depression since their content in l-tryptophan increases serotonin levels, more commonly known as the “happy hormone” in the brain [18]. In this updated and comprehensive review, the latest data on the anticancer activity of cucurbitacins, targets and molecular mechanisms of action, and perspectives in chemotherapeutic therapy by association with other agents are synthesized. In the light of the encouraging results obtained from preclinical pharmacological studies, new windows are opening for the generation of new ideas and strategies for cancer chemotherapy.

## Review methodology

Data on the anticancer effects of cucurbitacins, necessary to carry out the current review were obtained by analyzing the following specialized databases PubMed/MedLine, TRIP database, Web of Science, Google Scholar, and ScienceDirect using the following MesH terms: “Antineoplastic Agents, Phytogenic/pharmacology”, “Animals”, “Apoptosis/drug effects”, “Cell Line, Tumor”, “Cell Transformation, Neoplastic/drug effects”, “Cucurbitacins/chemistry”, “Cucurbitacins/pharmacology”, “Cucurbitacins/therapeutic use”, “Drug Design”, “Drug Therapy/Combination” “Humans”, “Molecular Targeted Therapy”, “Neoplasms/drug therapy”, “Neoplasm Invasiveness”, “Neoplasms/pathology”, “Phytotherapy”, “Signal Transduction/drug effects”. The study included the published papers with full text that highlighted the mechanisms and anticancer signaling pathways of cucurbitacins resulting from preclinical pharmacological studies. Papers with duplicate titles, abstracts and pharmacological experiments that included homoeopathic preparations associated with cucurbitacins were excluded. The scientific names of plant species were validated following WorldFloraOnline [19] and chemical formulas according to ChemSpider [20]. The most representative data were summarized in tables and figures.

## Cucurbitacins: origin, traditional uses and therapeutic potential

Among living organisms, some of them exhibit secondary metabolites unique to some species being considered chemotaxonomic markers because they reveal an evolutionary relationship [21]. Nevertheless, some other metabolites occur in organisms without taxonomic similarity, which is related to specific roles in nature. Hence, environment-mediated stimuli probably lead to the emergence of metabolites in non-related species [22]. Cucurbitacins belong to the latter classification, representing an intriguing convergence between plants, fungi and even some animals. These stimuli can be authenticated with an analogous ecological role—their intensive bitterness is toxic to a wide range of herbivores, microorganisms, insects, and parasites, thus protecting their hosts from predators [23]. Cucurbitacins are more commonly present within the plant kingdom, with the vast majority being found in the Cucurbitaceae family, although they can also exist in several other taxonomically distant families. Nonetheless, some studies have also reported the presence of cucurbitacins in both the fungi and the animal kingdom. Therefore, establishing the distributions of secondary metabolites in this family is of utmost importance.

Cucurbitacins are mainly found in the Cucurbitaceae family, which consists of 965 plant species in 120 genera. Most of them are consumed in vegetables or in fruits, but they also show medicinal value, the reason why they have been used for traditional medicine in China [24]. Cucurbitacins within this family are more commonly present in *Cucumis sativus* (cucumber), *Cucurbita moschata* (pumpkin), *Citrullus lanatus* (watermelon), *Cucumis melo* (melon), *Cucurbita pepo* (squash), *Lagenaria siceraria* (bottle gourd), *Luffa acutangula* (ridge gourd), and *Bryonia dioica* (red/white bryony). Furthermore, a special kind of cucurbitacins from the Cucurbitaceae family called momordicosides can be found in the *Momordica charantia* (bitter melon or African cucumber). Cucurbitacins have diverse therapeutically important and attractive bioactivities, including in cancer chemoprevention/chemotherapy, and numerous preclinical studies have proven these properties (Table 1) [25–27].

**Table 1:** Summarized data regarding the botanical sources and potential pharmacological effects of cucurbitacins

Cucurbitacins/Sources	Pharmacological effects	Refs.
*Cucumis sativus* (Cucumber)	Anticancer potential against liver cancer HepG2 cell	[28]
*Cucurbita moschata* (Butternut Squash)	Anticancer potential ↓cells tumour growth ↑ribosome-inactivating proteins	[17, 29, 30]
*Cucumis melo* (Melon)	Analgesic, anti-inflammatory, antioxidant, antiulcer, anticancer, antimicrobial, diuretic, antidiabetic, hepatoprotective, immunomodulator	[17, 29, 30]
*Citrullus lanatus* (Watermelon)	Antidiabetes	[33]
*Cucurbita pepo* (Pumpkin)	Antioxidant, anti-inflammatory, antiviral, antimicrobial, analgesic Anti-carcinogenic, anti-proliferative Pro-apoptotic properties against tumor cells	[17, 34]
*Iberis amara* (Wild candytuft)	Anti-inflammatory	[39, 40]
*Coutarea hexandra* (Coutarea)	Treatment of malaria, cancer, inflammation, and diabetes	[15]
*Begonia nantoensis*	Cytotoxic effect against cancer cell lines	[43]
*Kageneckia oblonga* (Bollen)	Analgesic, antipyretic, anti-inflammatory	[44, 45]
*Rubus chingii* (Chinese raspberry fruit)	Anti-ageing, anticancer, antioxidant, anti-inflammatory and antidiabetic	[46]
*Russula lepida* (Russula)	Antitumor activity	[50]
*Dorid nudibranchs*	Cytotoxicity against cancer cells	[54]

The extract from *Cucumis sativus* flowers was isolated and tested for their anticancer potential against liver cancer HepG2 cell line. With a LD_50_ of 103.7 µg/mL, this extract induced apoptosis in the HepG2 cell line [28].

Hou et al. evaluated the anticancer potential of *Cucurbita moschata* in an in vitro model, using K562 human leukaemia cells, B16 murine melanoma cells, and A549 lung adenocarcinoma cells. They confirmed that isolated compounds from this vegetable inhibit cell tumour growth by working like ribosome-inactivating proteins [17, 29, 30]. *Cucumis melo* is well known for its beneficial pharmacological assets, such as analgesic, anti-inflammatory, antioxidant, antiulcer, anticancer, antimicrobial, diuretic, antidiabetic, hepatoprotective, and immunomodulator [31]. Vella et al. have recently established the significance of seeds and peels from *Cucurmis melo*, which until then were only considered waste. They concluded that the *Cucumis melo* by-products are a source of polyphenols and tannins, responsible for antioxidant and anticarcinogenic properties, respectively. Hence, the valorization of this entire vegetable is extremely important because it can reduce waste, as well as its environmental impact and the economic costs associated with its disposal. Besides, it can also be a source of active compounds for cosmetics, and pharmaceutical products [17, 32]. *Citrullus lanatus* has a potential use in the treatment of diabetes mellitus being investigated in vivo with obese and diabetic-induced rats. Watermelon extracts seems to play an important role in the prevention of diabetes’s complications, through attenuation of specific parameters in the kidneys and liver of diabetic animals [33].

*Cucurbita pepo* has been used in traditional medicine in several countries, treating patients infected with worms and parasites. In Europe, it has helped in the treatment of prostate enlargements, as well as irritable bladders. Moreover, it has also been used due to its antioxidant, anti-carcinogenic, anti-inflammatory, antiviral, antimicrobial, and analgesic properties. The oil obtained by cold pressure from *Cucurbita pepo* seeds is rich in antioxidant and antimicrobial components. Thus, it can be useful in some cosmetic formulations as it protects against dermatological wounds [17, 34]. There have been some reports regarding the cytotoxicity activity of *Cucurbita pepo*. Wang et al. detected a dose-dependent inhibitory effect between *Cucurbita pepo* fruit extracts and HeLa and HepG2 cell growth [35]. Martinez et al. observed in HL60 tumour cells, that it inhibits significantly the H_2_O_2_-induced damage and presents anti-proliferative and pro-apoptotic properties [36]. Besides the Cucurbitaceae family, cucurbitacins are also present in other plant families and some fungi and animals. Below follows some of these examples.

One of the most extensive plant families distributed worldwide is Brassicaceae, which comprises almost 400 genera and 4000 species. Interestingly, this family used to be known as Cruciferae owing to their cruciform appearance and flower shape [37]. A vast majority of the several Brassicaceae species are vegetables, which are commonly identified by their functional properties, like their phytochemical composition. The phytochemicals are categorised as micronutrients, macronutrients, and secondary metabolites. There are currently some reports linking a wide spectrum of bioactivity of these secondary metabolites to the prevention and treatment of several chronic diseases, such as obesity, type 2 diabetes, cardiovascular diseases, cancer, and osteoporosis. Furthermore, antioxidant activity, as well as antimicrobial capacity have also been detailed. This phenomenon is mainly on account of the synergistic effect of glucosinolates, polyphenols, and triterpenes (specific cucurbitacins), the main constituents in cruciferous plants [38]. Khayyal et al. evaluated the anti-inflammatory and antioxidant properties of *Iberis amara* extracts (rich in cucurbitacins) in rats, making use of both acute and chronic experimental models of inflammation. A dose-dependent reduction in inflammation was observed in both models, reflecting the extract's potent anti-inflammatory properties. Furthermore, other biological roles have also been assigned to *Iberis amara* (due to the presence of cucurbitacins B, E, and I), such as antifeedant activity, protecting many brassicaceous species [39, 40].

*Picria fel-terrae, Neopicrorhiza scrophulariiflora and Gratiola officinalis* belong to the family Scrophulariaceae, and have been used in traditional medicine, due to their cucurbitacins' content. [14, 41, 42]. *Coutarea hexandra*, from the Rubiaceae family, has been used traditionally in the treatment of malaria, inflammation, and diabetes. Olmedo et al. performed, with cucurbitacins isolated from the ethanolic extract (80%) of this plant, sulphorhodamine B assay in the following cancer cell lines: breast (MCF-7), lung (H-460) and central nervous system (SF-268), observing moderate cytotoxicity [15]. Wu et al. isolated some cucurbitacins from *Begonia nantoensis* (Begoniaceae family) such as cucurbitacin B, dihydrocucurbitacin B, cucurbitacin E, dihydrocucurbitacin E, and cucurbitacin I and evaluated their cytotoxicity. These compounds presented strong cytotoxic effects against the following cancer cell lines: gastric (NUGC-3), nasopharyngeal (HONE-1), breast (MCF-7) and lung (A549) [43].

Stems and leaves of *Kageneckia oblonga* (Rosaceae family) containing cucurbitacin’s derivatives have analgesic, antipyretic, and anti-inflammatory activity [44, 45]. East Asian countries commonly use *Rubus chingii* s unripe fruits to treat several diseases, especially those related to kidney deficiencies. Pharmacological studies validated the anti-ageing, anticancer, antioxidant, anti-inflammatory and antidiabetic properties due to its content in cucurbitacins [46]. *Aquilaria agallocha* (Thymelaeaceae family) is one of the largest producers of agarwood used throughout history in religious ceremonies, herbal medicines and perfumes, and whose composition is rich in cucurbitacins particularly E and I) [47]. Cucurbitacins have also been isolated from a few genera of mushrooms, including *Hebeloma vinosophyllum*, *Russula lepida*, and *Leucopaxillus gentianeus*. Several studies reported that cucurbitacins exert a protective role against mushroom predators, such as microorganisms and insects, due to their cytotoxicity. For example, the reason why the *Hebeloma vinosophyllum* is a poisonous mushroom is believed to be due to the presence of hebevinosides I–XI, together with hydroxyhebevinogenin and methoxyhebevinogenin, some derivatives from cucurbitacins [48, 49]. *Russula lepida* (Russulaceae family) has already been exploited throughout the years as both food and medicinal agents in China due to its content in seco-cucurbitacins—at least three seco-cucurbitacins were already isolated. Specifically, the extracts of *Russula lepida*’s fruiting bodies exhibited antitumour activity [50]. Furthermore, they also demonstrated a protein tyrosine phosphatase 1B (PTP1B) inhibitory activity—a compound manipulated as a negative regulator in the signal transduction via insulin and leptin pathways—without showing cytotoxicity [51, 52].

The mushroom* Leucopaxillus gentianeus,* to protect itself from external predators, first accumulates inactive fatty acid esters, such as 16-oleyl, 16-linoleyl, and 16-palmityl esters in its tissues. Then, after injury, some lipases cleave these esters, transforming them into a more active compound—the bioactive metabolite cucurbitacin B, warding off external attacks due to its toxicity [23, 53]. The shell-less marine molluscs (*Dorid nudibranchs*) are particularly susceptible to predators and as a chemical defence mechanism, they release terpenoid metabolites—particularly cucurbitacins. Interestingly, these cucurbitacins showed modest in vitro cytotoxicity against human ovarian carcinoma (HEY) and human glioblastoma/astrocytoma (U373) cell lines [54].

## Cucurbitacins in anticancer pharmacological research: molecular mechanisms of action and efficacy

One of the fundamental characteristics of cancer cells is probably the ability to permanently stimulate their growth and proliferation [55]. Cancer cells have a low dependence on external proliferative stimuli and often do not need stimulation to multiply; through the mutations of some oncogenes, these cells acquire a proliferative autonomy, producing their mitogenic signals [2, 8, 56]. The major strategies used by tumor cells to achieve their proliferative independence are the following: the production of their growth factors; dysregulation of growth factor receptors, which transduce proliferative signals inside the cell; alteration of components of cytoplasmic signaling pathways, which produce a flow of mitogenic signaling without their stimulation by receptors; for example, the MAP-kinase (mitogen-activated protein-kinase) pathway, consisting of the proteins: RAS, RAF, MEK, MAPK (mitogen-activated protein kinase), ERK (extracellular signal-regulated kinase), FOS, plays a central role in human cancers [9, 57, 58]. The different cucurbitacins have been studied in experimental preclinical pharmacological studies and found to possess anticancer properties for a myriad of cancer types (Table 2).

**Table 2:** The anticancer mechanisms of cucurbitacins tested in vitro and in vivo experimental cancer models

Tested cucurbitacin	Experimental model	Anticancer mechanisms	Refs.
Cucurbitacin B	In vitro Melanoma cells A375	↓proliferation ↓MAPK	[79]
	In vivo Mice NOD-SCID xenograft melanoma model	↑ cytotoxicity ↓tumour growth	
	In vitro Gastric cancer cells SGC7901/DDP	Reversed multi-drug resistance ↓HIF-1a, ↓P-gp ↑apoptosis, ↑autophagy ↓CIP2A/PP2A/mTORC1, ↓mTORC1, ↓PP2A	[80]
	In vitro Hepatoma human cells HepG2, BEL-7402	↓cell invasion, ↓migration ↓MMP-9,↓ ERK 1/2,↓ p38, ↓Akt	[81]
	In vitro Non-small cells lung cancer (NSLC) H1299	↑epigenetic modifications ↓histone deacetylase ↓DNA methyltransferase ↑tumour suppressor genes: ↑CDKN1A, ↑CDKN2A ↓oncogenes ↓β-catenin, ↓Wnt3, ↓Wnt3a	[82, 83]
	In vivo Mice Lung tumourigenesis model	↓angiogenesis	
	In vitro Human cholangiocarcinoma cells KKU-100	Antiproliferative Cell cycle arrest at the G2/M phase ↓cyclin A, cyclin D1 ↑p21, ↑p53, ↓FAK, ↓EGFR, ↓HER2 ↓FAK/PI3K/PDK1/AKT, FAK/p53	[84]
	In vitro Breast cancer cells MDA-MB-231, SKBR3- MCF-7, 4T-1	↓ cells growth ↓HER2, ↓EGFR, ↓ILK1/ YB-1/Twist ↓ITGA6, ↓ITGB4	[85]
	In vivo Mice MDA-MB-231 athymic nude model	↓tumour volume	
	In vitro Breast cancer cells MDA-MB-231 cells	↑apoptosis, ↑morphologic changes ↑irregular polymerization of the microtubule ↑arrest at the G2/M cell cycle phase ↓c-Myc, ↓nucleophosmin/B23	[86]
	In vitro Breast cancer cells MDA‐MB‐231, SKBR‐3	↓cell migration ↓RAC1/CDC42/RhoA ↓FAK, ↓RAC1, ↓CDC42	[87]
	In vivo Mice BALB/c nude bearing SKBR‐3 cells	↓metastasis	
	In vitro Prostate cancer cells LNCaP, PC-3	↑apoptosis ↑Caspase 3, ↑ Caspase 7 ↑Sub-G0/G1 phase	[88]
	In vivo Mice PC-3 xenograft growth model	↓ATP citrate lyase (ACLY)	
	In vitro Human glioblastoma cells line U87	↓angiogenesis ↓α5β1	[69]
Cucurbitacin D	In vitro Cervical cancer cells CaSki, SiHa	↓tumor cells growth, ↓metastasis ↑annexin V, ↑ PARP ↑cell cycle arrest at the G1/S phase ↑CDK4, ↑cyclin D1 ↓RB protein phosphorylation ↓PI3K/AKT, ↓STAT3, ↓MMP9, ↓c-Myc	[70]
	In vivo Athymic nude mice bearing CaSki cells	↓ tumour growth	
	In vitro Pancreatic cancer cells AsPC-1, Capan-1	↑apoptosis ↑cell cycle arrest at the G2/M phase ↑ROS, ↑p38, ↑c-Jun, ↑ROS/p38 ↓cyclin B1, ↓PARP ↓phospho-cdc2, ↓phospho-cdc25c ↑p21, ↓cyclin/CDK ↑caspases 7, ↑caspase 8	[91]
	In vitro Non-small cells lung cancer NSCLC-N6 cells	cell cycle arrest at the G1 phase ↑CDK1 mRNA ↓p53	[72]
Cucurbitacin E	In vitro Glioblastoma cells GBM8401, U-87-MG	↓cell proliferation, ↑apoptosis ↑ G2/M phase of the cell cycle ↓mitosis, ↓CDC2, ↓cyclin B1 ↓GADD45β, ↓CDC2/cyclin B1	[73]
	In vitro A549 non-small cells lung cancer NSCLC cells lung cancer cells	↑apoptosis ↑EGFR, ↑EGFR/MAPK, ↑ERK1/2, ↓MEK1/2 ↑caspases -3, ↑caspase -9, ↓survivin, ↓STAT3 ↓cyclinA2, ↓cyclinB1, ↓cyclin E1 ↑G1/G0 phase of the cell cycle	[74]
	In vitro GBM8401malignant glioma cells GBM8401	antiproliferative, ↓cell growth ↑cell cycle arrest at the G2/M phase ↑GADD45γ, ↓B1/CDC2	[75]
Cucurbitacin I	In vitro SKOV3 ovarian cancer cells PANC-1 pancreatic cancers cells	↑apoptosis, ↑ autophagy ↑ERS, ↑IRE1α, ↑PERK, ↑caspase-12 ↑CHOP, ↑Bax	[76]
	In vitro colon cancer cells SW480	↓cell viability, ↑apoptosis, ↓proliferation ↑cell cycle arrest at the G2/M phase ↓cyclin A, ↓cyclin B1, ↓CDC25C, ↓CDK1 ↓ CDK1/cyclin B1, ↓caspases -3, -7, -8, -9	[77]
	In vivo Mice Syngeneic transplanted CT-26 BALB/c	↓tumour growth ↓proliferation	
Cucurbitacin II	In vitro Non-small cells lung cancer NSCLC lung cancer cells A549	↑apoptosis, ↑cell cycle arrest ↓EGFR, ↓EGFR/MAPK, ↓cyclinB1, ↓ERK1, ↓MEK1, ↓MEK2, ↑survivin, ↑BRAF, ↑Raf1, ↑ERK2, ↑STAT3, ↓ERK1, ↓MEK1	[78]
		↑apoptosis, ↓STAT3 ↑G2/M phase cell cycle arrest, ↓EGFR/MAPK	[79]
		↓cell growth, ↑apoptosis ↓ STAT3, ↓EGFR	[80]

↑ increase; ↓decrease; MAPK: mitogen-activated protein kinase; HIF-1a: hypoxia-inducible factor 1-alpha; P-gp: P-glycoprotein; CIP2A: cellular inhibitor of PP2A; PP2A: protein phosphatase 2; mTORC1: mammalian target of rapamycin complex 1; MMP-9: matrix metallopeptidase 9; ERK 1/2: extracellular signal-regulated kinases 1 and 2; p38: group of MAPK; Akt: protein kinase B; CDKN1A: cyclin dependent kinase inhibitor 1A; CDKN2A: cyclin dependent kinase inhibitor 2A; Wnt3: proto-oncogene family member 3; Wnt3a: proto-oncogene family member 3a; p21: cyclin dependent kinase inhibitor; p53: tumor suppressor gene; FAK: focal adhesion kinase; EGFR: epidermal growth factor receptor; HER2: human epidermal growth factor receptor 2; PI3K: phosphoinositide 3-kinase; PDK1: 3-phosphoinositide-dependent kinase 1; ILK1: integrin-linked kinase 1; YB-1: Y-box binding protein; Twist: helix-loop-helix transcription factor; ITGA6: integrin subunit alpha 6; ITGB4: integrin subunit beta 4; c-Myc: multifunctional transcription factor; nucleophosmin/B23: multifunctional nucleolar protein; RAC1: Ras-related C3 botulinum toxin substrate 1; CDC42: cell division control protein 42; RhoA: Ras homolog family member A; ACYL: ATP citrate lyase; α5β1: alpha 5 beta 1 protein; PARP: poly (ADP-ribose) polymerases; CDK4: cyclin dependent kinase 4; RB: retinoblastoma protein; STAT3: signal transducer and activator of transcription 3; ROS: reactive oxygen species; c-Jun: transcription factor Jun; phospho-cdc2: phosphorylated cell division cycle 2; phospho-cdc25c: phosphorylated protein that regulates cell division; CDK1: cyclin dependent kinase 1; GADD45β: growth arrest and DNA damage-inducible 45 beta; MEK1/2: mitogen activated protein kinase kinases 1 and 2; GADD45γ: growth arrest and DNA damage-inducible 45 gamma; B1/CDC2: cyclin B1-dependet Cdc2 kinase; ERS: endoplasmic reticulum stress; IRE1α: inositol-requiring transmembrane kinase endoribonuclease 1 alpha; PERK: endoplasmic reticulum kinase; CHOP: DNA damage-inducible transcript 3 protein; Bax: bcl-2-like protein 4; CDC25C: cell division control protein 25C; survivin: member of the inhibitor of apoptosis gene family; BRAF: B-Raf proto-oncogene; Raf1: Raf-1 proto-oncogene

### Cucurbitacin B

The anticancer activity of cucurbitacin B, obtained from the dried rhizome powder of *Corallocarpus epigaeus*, was determined to induce the apoptotic machinery in the A375 melanoma cell line, by targeting the MAPK pathway and suppressing proliferation. This cytotoxic effect was confirmed in vivo using a xenograft melanoma model, in male NOD-SCID mice induced by A375 cells, with cucurbitacin B substantially reducing tumour growth when compared with untreated mice [59]. Similarly, for gastric cancer, cucurbitacin B reversed the multi-drug resistance of the SGC7901/DDP gastric cancer cells by downregulating the drug-resistant protein HIF-1a and P-pg. It also promoted apoptosis and autophagy via modifications of the CIP2A/PP2A/mTORC1 signaling axis. In detail, cucurbitacin B induced apoptosis and autophagy by inhibiting mTORC1. This inhibition is dependent on PP2A activity, and this study showed that the triterpenoid may target CIP2A to reactivate PP2A [60].

Cucurbitacin B was also found to suppress cell invasion and migration induced by 12-*O*-tetradecanoylphorbol 13-acetate (TPA) of the hepatoma human cell lines HepG2 and BEL-7402. The therapeutic molecule additionally inhibited the metabolic activity of TPA-induced MMP-9, by inactivating the extracellular signal-regulated kinase (ERK) 1/2, p38, and the Akt signaling pathway [61].

For non-small cell lung cancer (NSCLC), another study reported that treatment with cucurbitacin B led to inhibition of histone deacetylase and DNA methyltransferase in the H1299 cells, and this inhibition in turn allows for epigenetic modifications, such as the upregulation of important tumour suppressor genes and downregulation of key oncogenes. Specifically, the triterpenoid induced gene expression of CDKN1A and CDKN2A and downregulated the tumour promoter gene, hindering cellular growth and promoting apoptosis. In vivo treatment with cucurbitacin B lowered tumour mass and incidence in a lung tumourigenesis model induced by 4-(methylnitrosamino)-1-(3-pyridyl)-1-butanone, decreasing tumour angiogenesis in a dose-dependent manner [62]. The same authors also found that cucurbitacin B can inhibit the expression and nuclear translocation of β-catenin, a key participator in cancer development and metastasis, by suppressing the expression of Wnt3 and Wnt3a ligands, commonly overexpressed in NSCLC [63].

On human cholangiocarcinoma cells, cucurbitacin B inhibited the growth and replication of KKU-100 cells, preventing colony formation. This antiproliferative ability is linked to cell cycle arrest at the G2/M phase, downregulation of cyclins A and D1, and upregulation of p21 and p53. In addition, the study found that cucurbitacin B inhibits the activation of folate adhesion kinase (FAK) in KKU-100 cells by possibly targeting the expression of EGFR and HER2, frequently found overexpressed in the disease, and thus acts as a modulator of the FAK/PI3K/PDK1/AKT and FAK/p53 signaling pathways [64].

Particularly regarding breast cancer, the anticancer potential of cucurbitacin B has been heavily documented, in both in vitro and in vivo studies. Of note, Cucurbitacin B was able to suppress breast cancer growth in four different cell lines, namely MDA-MB-231, SKBR3- MCF-7 and 4T-1, by reducing the expression of HER2 and EGFR in a dose-dependent pattern. The HER2 inhibition was higher in the cell lines where the ILK1/YB-1/Twist signalling axis, identified as a regulator of HER2 expression, was also inhibited. Furthermore, when compared with other integrins, the triterpenoid compound significantly suppressed ITGA6 and ITGB4 expression, commonly overexpressed in breast cancer, in a time-dependent way. In vivo testing using MDA-MB-231 injected orthotopically in female athymic nude mice showed up to 50% tumour volume reduction following cucurbitacin B treatment when compared with untreated mice. In the 4T-1 orthotopic model, injected in BALB/c mice, the treated group had about 40% less tumour volume than the control mice [65]. A different study found that cucurbitacin B disrupts the cytoskeletal network of MDA-MB-231 cells, resulting in fast morphologic changes and irregular polymerization of the microtubule network. These alterations lead to apoptosis increase and cause arrest at the G2/M cell cycle phase. Additionally, cucurbitacin B treatment significantly reduced the c-Myc and nucleophosmin/B23 expression, with an increased translocation of the latter phosphoprotein from the nucleolus to the nucleoplasm, thereby preventing its cytoplasmic shuttling and subsequent role in cell proliferation [66]. Furthermore, cucurbitacin B acts as a mediator in the distribution and reorganization of cytoskeletal proteins via RAC1/CDC42/RhoA signaling, altering the mechanical properties of MDA‐MB‐231 and SKBR‐3 breast cancer cells. Cucurbitacin B treatment inhibited FAK and vinculin expression, affecting the cytoskeleton’s cell adhesion and contractility. It also interfered with the activity of the GTPases RAC1 and CDC42, which are important regulators of cell tension, thereby impacting cell migration in vitro and in vivo breast cancer models. Further in vivo testing on BALB/c nude mice bearing SKBR‐3 cells suggest that cucurbitacin B suppresses breast cancer metastasis to the lungs and liver [67].

For prostate cancer, cucurbitacin B showed anticancer activity, associated with apoptosis induction. This is evidenced by a substantial increase in the activity of Caspase 3/7 in the human prostate cancer cell lines LNCaP and PC-3, as opposed to normal prostate epithelial cells (PrEC), an increase in the Sub-G0/G1 phase, but also cleavage of Poly (ADP-ribose) polymerase, involved in DNA repair. In vivo, cucurbitacin B pre-treatment inhibited considerably PC-3 xenograft growth in athymic mice compared to controls after 31 days. The authors proposed cucurbitacin’s dose-dependent inhibition of ATP citrate lyase, or ACYL, as the anticancer mechanism of cucurbitacin, in in vitro and in vivo prostate tumour models [68].

On the human glioblastoma cell line U87, cucurbitacin B isolated from the leaves of *Ecballium elaterium* exhibited integrin-associated anticancer activity. In-depth, the article states that the molecule conditioned U87 cells from migrating and adhering to fibronectin dose-dependently, by targeting the α5β1 glioma protein and disrupting its activity, resulting in decreased motility and directional persistence of the tumour cells. This particular integrin plays a role in tumour formation and angiogenesis, and cucurbitacin B was able to inhibit angiogenesis in human microvascular endothelial cells, dose-dependently, in concentrations as low as 100 nM [69].

### Cucurbitacin D

For cervical cancer, cucurbitacin D was able to decrease tumour cell growth and metastasis, dose-dependently, in the CaSki and SiHa cell lines by inducing apoptosis, measured by increased Annexin V staining and PARP protein cleavage and causing cell cycle arrest at the G1/S phase by interfering with the cell cycle regulatory proteins CDK4 and cyclin D1, as well as preventing RB protein phosphorylation. Furthermore, this study suggests that cucurbitacin D acts as an inhibitor of the signalling pathway PI3K/AKT by targeting HPV E6, STAT3 and its downstream targets MMP9 and c-Myc. In vivo testing in athymic nude mice bearing CaSki cells determined that administering cucurbitacin D intratumorally inhibited the growth of the orthotopic xenograft tumours [70].

For pancreatic cancer, treatment with cucurbitacin D caused cell cycle arrest at the G2/M phase, apoptosis induction, and intracellular ROS production in the AsPC-1 and Capan-1 cell lines. Cucurbitacin D suppressed cyclin B1, phospho-cdc2, and phospho-cdc25c, while upregulating the expression of p21, a cyclin/CDK complex inhibitor. By activating caspases-7 and -8 and cleaving PARP, the molecule induced apoptosis. Furthermore, the generation of ROS was linked to phosphorylation of p38 and c-Jun. Together, the article proposes that these anticancer mechanisms were caused via the ROS/p38 pathway [71].

Regarding NSCLC, cucurbitacin D proved to be cytostatic on NSCLC-N6 cells, an action associated with cell cycle arrest at the G1 phase. Treatment with the molecule led to an overexpression of CDK1 mRNA, which accumulates during the G1 phase, increasing the activity of CDK1 over the intermediate level necessary for the G1/S transition. Additionally, the cell line used in the study has mutated and inactive p53, a protein that negatively regulates CDK1 transcription [72].

### Cucurbitacin E

The anticancer potential of cucurbitacin E against glioblastoma was investigated. The study demonstrates the compound’s ability to hinder cell proliferation and survival on the GBM8401 and U-87-MG cell lines, in a concentration-dependent manner. Cucurbitacin E treatment also led to an accumulation of cells at the G2/M phase of the cell cycle, therefore delaying mitosis. The cell cycle arrest was achieved by downregulating the expression of CDC2 and cyclin B1 proteins, disassociating the CDC2/cyclin B1 complex through GADD45β upregulation [73].

A more recent article found that cucurbitacin E was able to induce apoptosis and cause cell cycle arrest on NSCLC cells through the EGFR/MAPK pathway. More precisely, following cucurbitacin E treatment, the A549 cells exhibited an increase in the cleaved caspases-3 and -9, both apoptosis regulators, while also decreasing the expression of the apoptosis inhibitor protein survivin and the levels of phosphorylated STAT3. Cucurbitacin E reduced cyclin A2, cyclin B1 and cyclin E1 levels, resulting in an accumulation of cells at the G1/G0 phase of the cell cycle. Finally, the article showed that the derivative increased the phosphorylation of EGFR, altering the phosphorylation levels of the downstream members ERK1/2 and MEK1/2, found upregulated and downregulated, respectively [74].

The antiproliferative activity of cucurbitacin E on the brain in the malignant glioma GBM8401 human cell line was evaluated. Cucurbitacin E treatment inhibited cell growth by inducing cell cycle arrest at the G2/M phase, linked to GADD45γ upregulation and dissociation of the complex cyclin B1/CDC2 in a dose-dependent manner [75].

### Cucurbitacin I

For ovarian and pancreatic cancers, the antitumour action of cucurbitacin was researched. Li’s work demonstrated that, in SKOV3 ovarian cells and PANC-1 pancreatic cells, treatment with the compound induced cells’ apoptosis associated with endoplasmic reticulum stress or ERS. Cucurbitacin I raised the levels of ERS through activation of enzyme IRE1α and the kinase PERK, leading to apoptosis induction dependent on the activation of the caspase-12 and CHOP pathways, and dependent on Bax increase. The excessive ERS levels additionally prompted autophagic cell death [76].

The antitumour potential of cucurbitacin I on colon cancer was the focal point of Kim and colleagues’ research published in 2014. The article states that, following cucurbitacin I treatment, the SW480 cells suffered cell viability and proliferation decrease in a dose-dependent pattern, presenting minimal effect on the normal cell line CCD-18Co. Following other derivatives, cucurbitacin I induced cell cycle arrest at the G2/M phase, associated with a decrease in the expression of cyclin A, cyclin B1, CDC25C, and CDK1 and subsequent inhibition of the CDK1/cyclin B1 complex. As for apoptosis induction, cucurbitacin I treatment was linked to an increase in the cleavage of caspases-3, -7, -8, -9. In vivo, the derivative repressed the growth and proliferation of a syngeneic transplanted CT-26 BALB/c mice tumour model, and as observed in vitro, an increase in the apoptosis-related proteins and a decrease in the expression of G2/M phase cell cycle-related proteins was detected [77].

### Cucurbitacin II

**Fig. 1:**
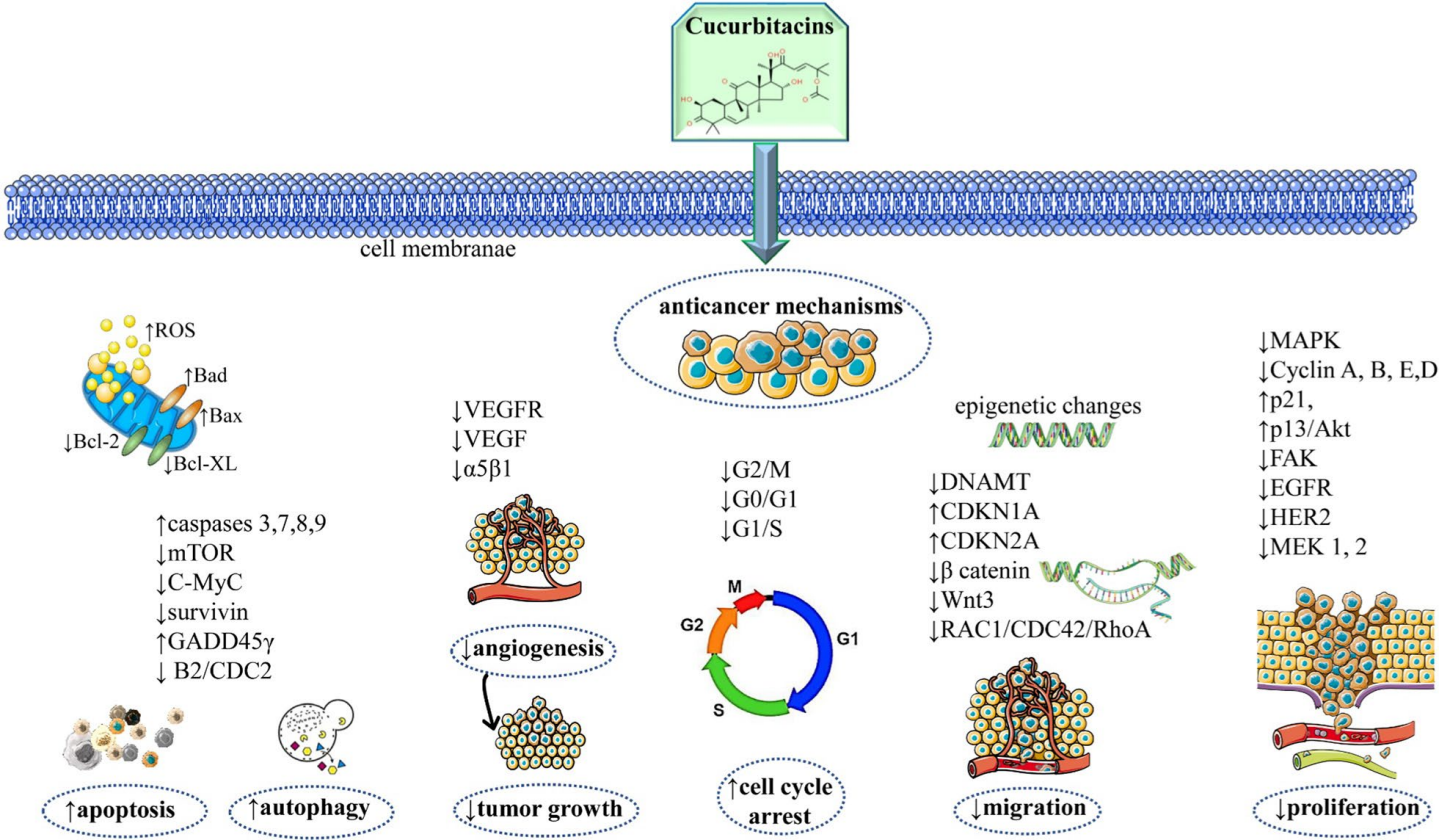
Summarized scheme with the most representative anticancer molecular mechanisms of cucurbitacins. ↑ increase, ↓decrease; Bax: Bcl-2-associated X protein; Bcl-XL: B-cell lymphoma-extra large; Bcl-2: B-cell lymphoma 2 protein; mTOR: mammalian target of rapamycin protein; VEGFR: vascular endothelial growth factor receptor; VEGF: vascular endothelial growth factor; DNAMT: mitochondrial DNA

The anticancer mechanisms of cucurbitacin IIa against NSCLC, namely its ability to induce apoptosis and cell cycle arrest, were investigated on the A549 cell line. The researchers found that cucurbitacin IIa acts as a tyrosine kinase inhibitor of the EGFR, and treatment with the molecule altered the expression of genes in the EGFR/MAPK pathway, namely downregulating the expression of cyclinB1, ERK1, MEK1, and MEK2, and upregulating the survivin, BRAF, Raf1, ERK2, and STAT3 genes. Notably, the expressions of ERK1 and MEK1 were significantly downregulated when compared with non-treated cells [78]. In 2021, a different group achieved corresponding results with cucurbitacin IIb in NSCLC research, namely acting as a tyrosine kinase inhibitor and CuIIb, inducing apoptosis via the STAT3 pathway and causing G2/M phase cell cycle arrest through the suppression of the EGFR/MAPK pathway [79]. Similarly, cucurbitacin IIb isolated from *Ibervillea sonorae* was tested as an antitumoural treatment option for NSCLC in the A549 cell line, reducing cell growth and inducing apoptosis at low concentrations and in a dose-dependent manner, while also inhibiting STAT3 expression and suppressing the activity of EGFR and/or of its downstream proteins [80]. Table 2 and Fig. 1 summarize the most representative anticancer mechanisms of cucurbitacins in different cancer modern experimental models.

## Synergistic anticancer effects of cucurbitacin B in combination with chemotherapeutic agents

Despite the numerous advantages of natural compounds in the treatment of several diseases, including cancer, some reports have found that combining these bioactive compounds with other clinically used chemotherapy drugs/small molecules strengthens the anticancer effect and reduces drug toxicity [81–83]. This combination therapy shows a promising treatment for cancer in both preclinical pharmacological studies in vitro and in vivo [84, 85]. In this section, an emphasis on the synergistic activity of the most used cucurbitacin—cucurbitacin B—with other compounds in several types of cancers is going to be detailed and the most important data are summarized in Table 3.

**Table 3:** The most important anticancer synergistic effects of cucurbitacin B in combination with chemotherapeutic drugs

Type of cancer	Type of chemotherapeutic agents combined with cucurbitacin B	Experimental model	Main results	Refs.
Breast cancer	Imatinib mesylate	In vitro MCF-7 cells	Anticancer synergistic effects ↓cell proliferation ↑apoptosis	[86]
	Gemcitabine docetaxel	In vitro MDA-MB-231 cells	Synergistic effects ↓cell proliferation, ↑apoptosis	[87]
		In vivo mice	↓tumour volume No signs of increased toxicity	
	Ionizing radiation	In vitro 4T1 cells	Blocks cancer cells in the G2/M phase ↑apoptosis, ↓cancer cells growth ↓p-STAT3, ↓c-Myc, ↓Bcl-2, ↓Bcl-xL, ↑caspase-9, ↑p21, ↑p53	[88]
Pancreatic cancer	SCH772984, an ERK inhibitor	In vitro HPAC cells	↑apoptosis, ↓cancer cells growth ↓EGFR, ↓PI3K/Akt/mTOR, ↓STAT3, ↑Bim, ↓McL-1, ↓Bcl-2, ↓Bcl-xl, ↓survivin, ↑cucurbitacin B sensitivity	[89]
		In vivo mice	↓ tumour growth	
Colorectal cancer	Gefitinib	In vitro HT-29, HCT-116 cells	Cell cycle inhibition ↑apoptosis, ↓EGFR, ↓JAK/STAT	[90]
	Imatinib mesylate	In vitro SW480 cells	↓cell proliferation ↑apoptosis ↓MMP-2, ↑degradation of extracellular matrix	[86]
Ovarian cancer	Cisplatin	In vitro A2780 cells	Cytotoxicity against the ovarian cancer cell line	[91]
	Paclitaxel	In vitro A2780/Taxol cells	Dose- and time-dependent cytotoxicity effect ↑G2/M phase arrest of the cell cycle, ↑p53, ↑p21, ↓Bcl-2, ↓caspase-3, ↓P-gp	[92]
Osteosarcoma	Methotrexate	In vitro human osteosarcoma cells	↓AKT, ↓mTOR	[93]

↑ increase, ↓decrease, JAK: Janus kinase; McL-1: induced myeloid leukaemia cell differentiation protein 1; Bim: Bcl-2-like protein 11

### Breast cancer

Despite recent advances in cancer therapies, breast cancer remains the second leading cause of death among women [94–97]. The combination of chemotherapeutic agents with non-chemotherapeutic agents is clinically interesting and plays a significant role in breast cancer management [83, 98]. By treating MCF-7 breast cancer cells with cucurbitacin B alone or in combination with imatinib mesylate, it was discovered that the combination treatment synergistically inhibited cell proliferation and induced apoptosis [86]. Similarly, combining gemcitabine or docetaxel with cucurbitacin B increases apoptosis, inhibiting the proliferation of MDA-MB-231 breast cancer cells in vitro. When analyzing these same compounds in vivo, the authors observed that the combination of either one of the drugs with cucurbitacin B significantly reduced tumour volume without any signs of increased toxicity [87]. Furthermore, cucurbitacin B combined with ionizing radiation blocks cancer cells in the G2/M phase and promotes apoptosis, inhibiting cancer cells. Additionally, this combination decreases the content of molecules such as p-STAT3, c-Myc, Bcl-2, and Bcl-xL and increases the content of apoptosis-related molecules such as caspase-9, p21 and p53 [88].

### Pancreatic cancer

In Western societies, pancreatic cancer is the fourth most common cause of cancer death, with a mortality rate of 95% within 5 years. This high mortality rate is primarily due to the insensitivity of pancreatic cancer to most chemotherapy and radiotherapy treatments. Zhou et al. combined cucurbitacin B and SCH772984, an ERK inhibitor, to inhibit pancreatic cancer cell growth and apoptosis by inhibiting the EGFR and its downstream signaling pathways, including PI3K/Akt/mTOR and STAT3. Additionally, they increased the pro-apoptotic protein Bim and reduced the anti-apoptotic proteins McL-1, Bcl-2, Bcl-xl, and survivin. By studying the synergistic combination of these two molecules in vivo, it was possible to observe a significant delay in tumour growth, both in the reduction of its volume and weight. Thus, the combined therapy of cucurbitacin B and SCH772984 resulted in critical growth inhibition of pancreatic cancer mice HPAC xenograft models. SCH772984 increased significantly cucurbitacin B sensitivity in pancreatic cancer cells but not in normal pancreatic ductal epithelial cells (used as a control) [89].

### Colorectal cancer

Colorectal cancer is the fourth most common cancer-related mortality in men and women worldwide [99–101] and associated bacterial and fungal infections can worsen the prognosis [102–105]. It is characterized by overexpression of EGFR and its downstream signaling pathways, such as Janus kinase/signal transducer and activator of transcription (JAK/STAT) [106, 107]. The combination of these two molecules results in significant apoptotic and anti-proliferative effects on HT-29 and HCT-116 colorectal cancer cell lines compared with cucurbitacin B alone [90]. By treating SW480 colorectal cancer cells with cucurbitacin B alone or in combination with imatinib mesylate, it was discovered that the combination treatment synergistically inhibited cell proliferation and induced apoptosis. Additionally, cucurbitacin B can also increase the inhibitory effect of imatinib mesylate on matrix metalloproteinase-2 (MMP-2) expression, a member of the MMP family, which is responsible for the degradation of extracellular matrix, in a dose-dependent manner [86].

### Ovarian cancer

One of the deadliest types of gynaecological cancer is ovarian cancer [108, 109]. In the first instance, platinum-based drugs, such as cisplatin, are used to treat the disease. Most patients, however, experience tumour recurrences that are resistant to cisplatin. Thus, co-administering chemotherapeutic agents with natural products may offer a synergistic effect [85]. El-Senduny et al. observed that cucurbitacin B exhibited cytotoxicity against the ovarian cancer cell line A2780, and pretreatment of cisplatin-resistant cell line A2780CP with this natural compound led to a significant increase in the cytotoxicity of cisplatin [91]. When investigating the effect of cucurbitacin B on human paclitaxel-resistant ovarian cancer A2780/Taxol cells, Qu et al. detected a dose- and time-dependent cytotoxicity. Furthermore, these cells were also blocked in the G2/M phase of the cell cycle, by several molecular mechanisms, such as upregulation of p53 and p21, downregulation of Bcl-2, activation of caspase-3 and suppression of P-gp [92].

### Osteosarcoma

Human osteosarcoma is the most common malignant bone tumour occurring in children and teenagers [8]. The estimated incidence rate worldwide is 4 million per year, with a peak incidence at the age of 15–19 years [110]. Owing to its numerous metastasis and high recurrence rate, its treatment requires several simultaneous approaches, such as surgery, radiotherapy, and chemotherapy. Thus, the combination of different compounds in the osteosarcoma treatment can be a potentially beneficial approach. Lee et al. showed that treatment with low doses of cucurbitacin B and methotrexate synergistically inhibit the AKT and mTOR signaling pathways in human osteosarcoma cells both in vivo and in vitro improving the tumour suppression rate [93].

## Limitation of the evidence

Cancer is a complex and dynamic disease, and its treatment requires substances with multiple actions, such as: detoxifying the body of harmful compounds (xenobiotics and endotoxins) with carcinogenic action; reducing oxidative stress; maintaining normal cell division; induction of malignant cell apoptosis; inhibition of angiogenesis; improving the antitumor immune response [5, 111–115]. The therapeutic anticancer potential of cucurbitacins depends on the quality and quantity of the bioactive compounds contained, the soil in which the plants are grown and the geographical location of these species. Adjuvant therapy must generally use purified and standardized extracts of active constituents that have a targeted pharmacological activity [116]. The standardization of cucurbitacins is necessary to ensure the identification, isolation and reproducibility of the active compounds in different pharmaceutical formulations. Another important limitation is the weak and variable absorption of cucurbitacins; as a result, new nano-formulations are needed to increase absorption, bioavailability and transport to the target in tumor cells [117, 118]. Also, the control of the therapeutic actions, the safety and the efficiency of the administration of the products must be ensured compared to the use of the whole plant or only some parts of the plant.

The main therapeutic limitation derives from the concerns associated with cancer treatment due to the aggressive nature of certain cancers and tumour resistance to chemotherapeutic drugs [119]. As previously mentioned, cucurbitacins have antiproliferative properties on several tumour cells, indicating their potential as anticancer agents. Furthermore, cucurbitacins synergistically enhance the efficacy of many other small-molecule drugs in cancer treatment. However, since most of the reported studies were performed in vitro, further in vivo work either on model animals or human clinical trials needs to be executed. All the findings presented in this comprehensive review suggest that cucurbitacins are potential candidates for use in combination therapy with clinical anticancer drugs.

## Conclusion and future perspectives

Cucurbitacins are structurally diverse triterpenes found in a wide range of plant families, especially the Cucurbitaceae and present several biological properties. Future research may benefit from chemical modifications of specific functional groups to improve pharmacokinetic and pharmacodynamic issues. Their anticancer potential has received increasing attention, as they can prevent the proliferation of different tumour cells by inducing apoptosis, cell cycle arrest, autophagy, and cytoskeletal disruption. These processes happen both in vivo and in vitro through multiple targets. Additionally, cucurbitacins exert strong synergistic anticancer effects when combined with clinically used chemotherapeutic drugs. New nanotechnological formulations must be developed to increase the bioavailability of cucurbitacins. Translational pharmacological studies are also necessary to establish the exact dose in humans and the best way of their administration. Further clinical studies on cucurbitacins should be performed to confirm their potential as drug candidates using these molecules from nature as new effective chemotherapeutics.

## Supplementary Information

**Additional file 1.** Cucurbitacins chemical characterization: identity, physical and chemical properties, isolation,synthesis of cucurbitacins and their derivatives.

## Data Availability

Not applicable.
