# Supplementary material for: Cucurbitacins as potential anticancer agents: new insights on molecular mechanisms
Source: J Transl Med. 2022 Dec 31;20:630. doi: 10.1186/s12967-022-03828-3 (PMC9805216; doi:10.1186/s12967-022-03828-3)
Supplement: Supplementary file 1 — Additional file 1. Cucurbitacins chemical characterization: identity, physical and chemical properties, isolation,synthesis of cucurbitacins and their derivatives. [file 12967_2022_3828_MOESM1_ESM.docx]

**Cucurbitacins chemical characterization**

**1. Identity**

(**Figure S1**). All cucurbitacins have a C-5/C-6 double bond, and most of them are tetracyclic but some present an extra ring due to formal cyclization between C-16 and C-24. The class of triterpenoids they belong to is characterized by the cucurbitane skeleton, 19-(10→9β)-abeo-10-lanost-5-ene, and presents several oxygen functions in different positions, such as keto, hydroxyl and acetoxy groups (Kaushik et al., 2015, Chen et al., 2012, Lang et al., 2012, Silva et al., 2016).


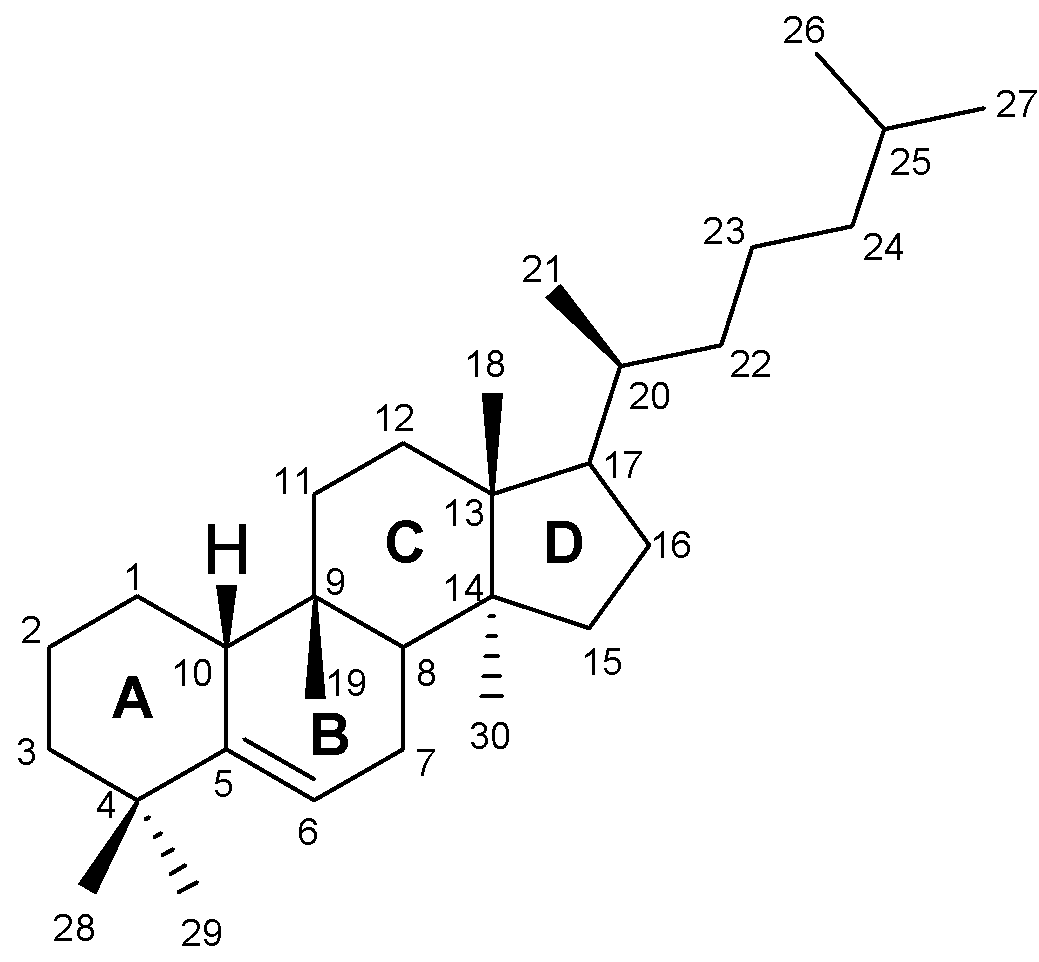


**Figure S1.** The basic skeleton of cucurbitacins/cucurbitane-type tetracyclic triterpenoids.

Unlike other groups of natural compounds, such as lanostanes that possess quaternary centres at C-10, C-13, and C-14 and maintain a relatively flat polycyclic network, cucurbitanes possess quaternary centres at C-9, C-13, and C-14 and have a cis-fused BC ring ending in a convex tetracyclic system (**Figure S2**) (Bucknam and Micalizio, 2022).


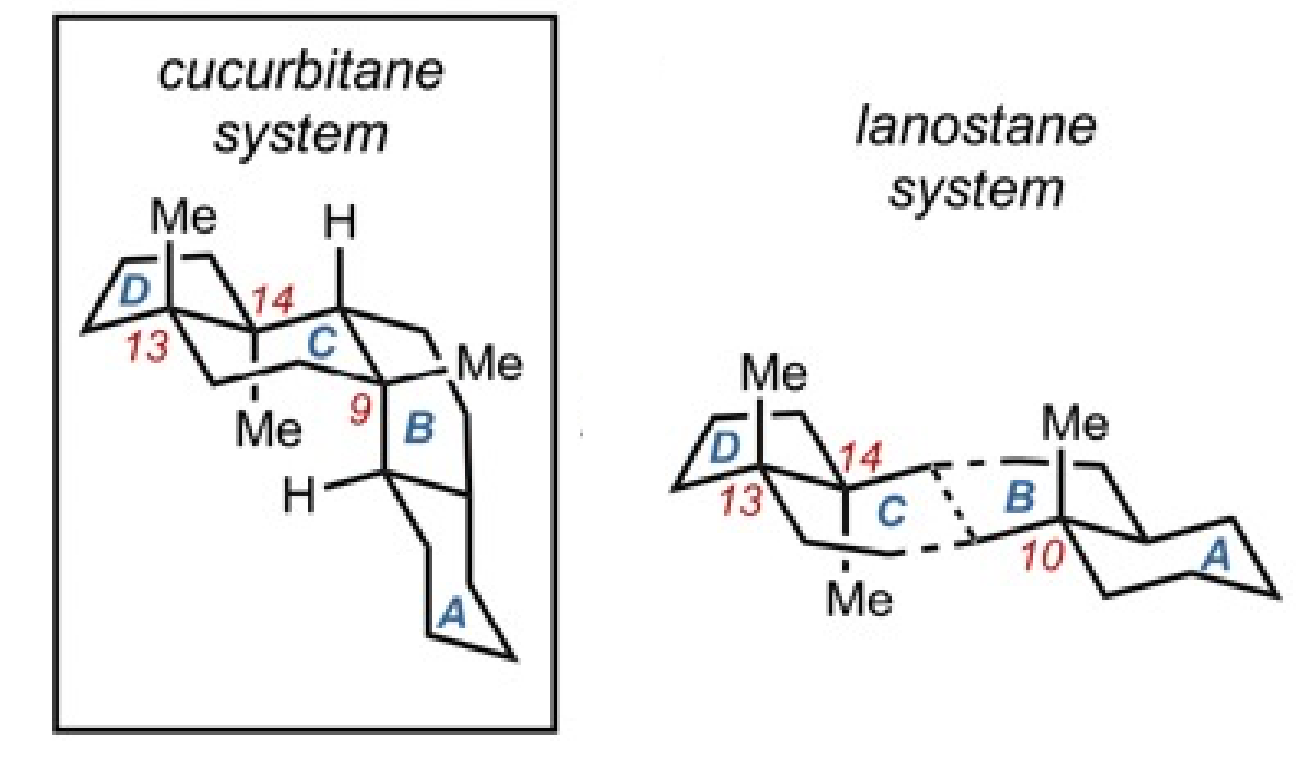


**Figure S2.** Cucurbitane system versus lanostane system. (From (Bucknam and Micalizio, 2022).

Cucurbitacins are organized according to the functional groups on rings A and C, the diversity in the side chain and stereochemical specifications. They are then divided into twelve categories designated by letters from A to T with over 200 derivatives based on isomerism, deoxidization, and dihydrogen (**Figure S3**). They can be found in the free or glycosylated one, and, in the latter, the saccharide usually is linked to C-2 (2-O-β-glycosides) (Kaushik et al., 2015, Alghasham, 2013, Lang et al., 2012, Samuel, 2019, Mir et al., 2019).


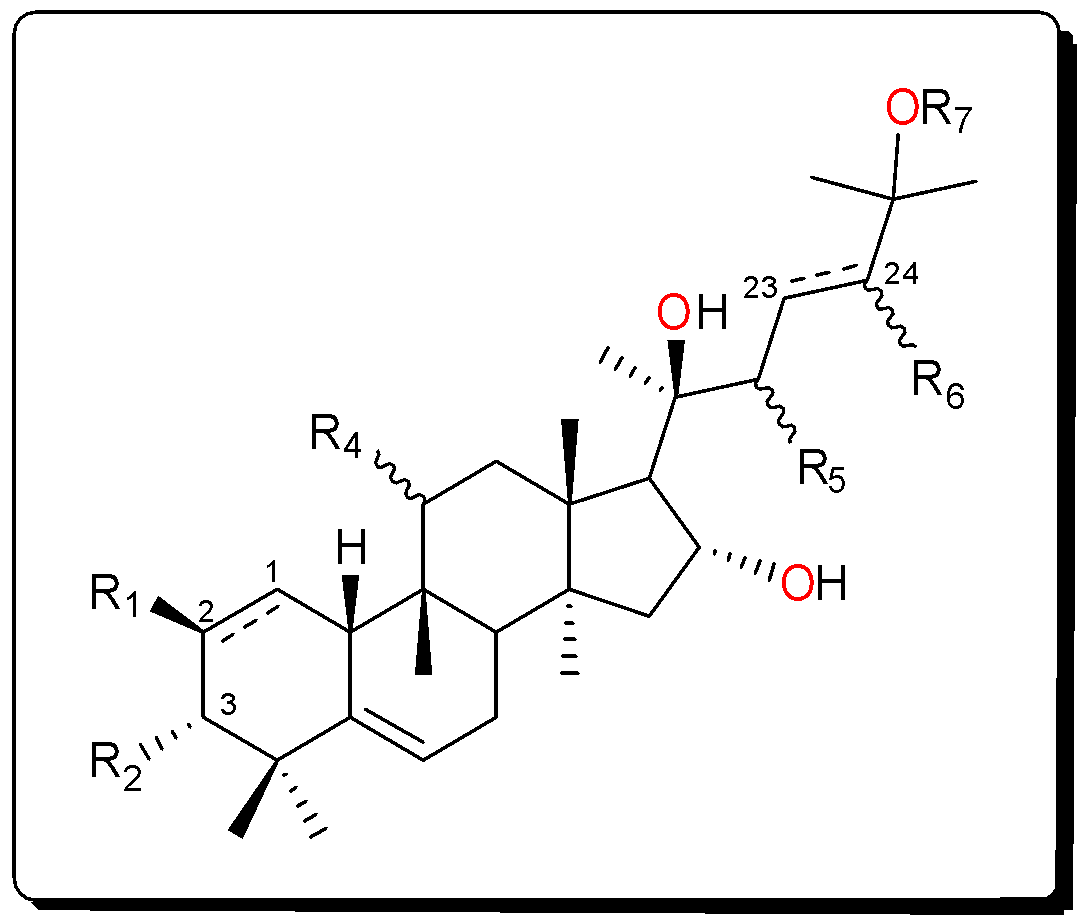


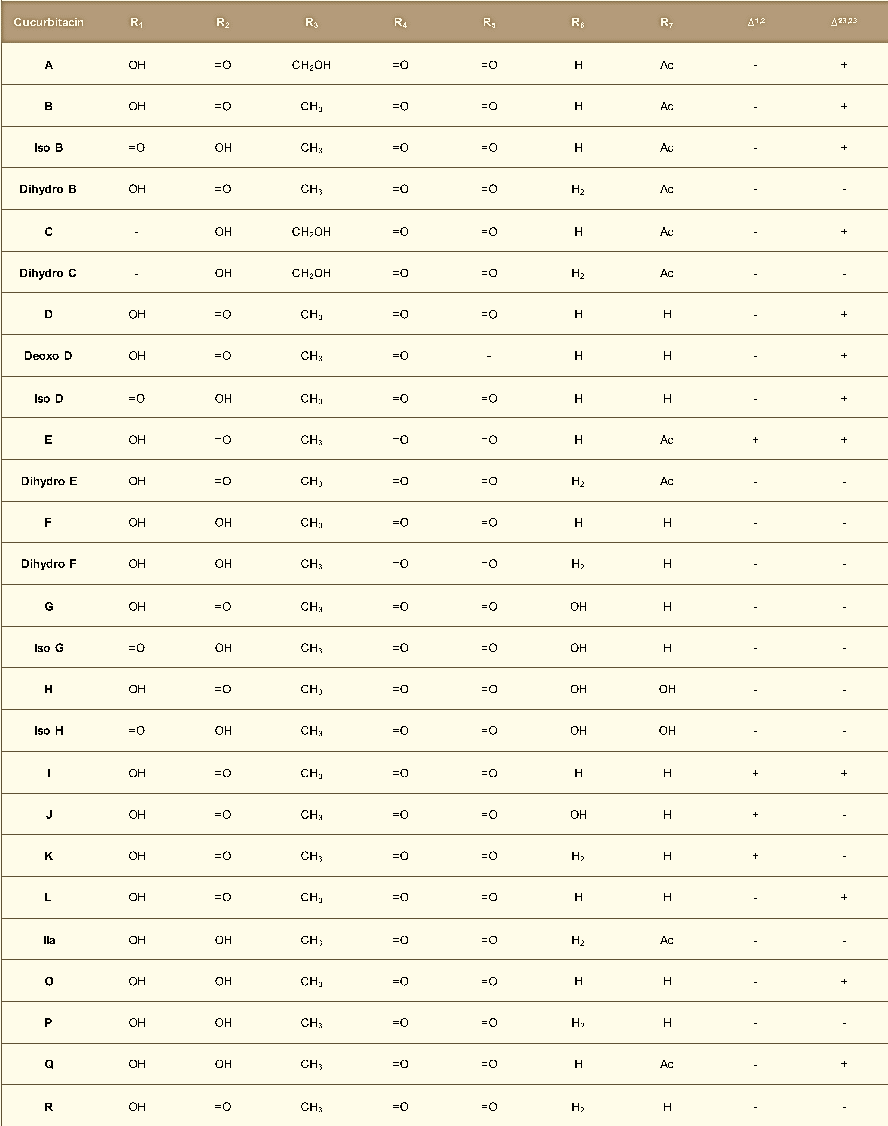


**Figure S3.** Main cucurbitacins and derivatives. As mentioned before, most Cucurbitacins are tetracyclic but cucurbitacins S and T have an additional ring. Cucurbitacins B, D, E, and I are the most widely used in *in vitro* and *in vivo* studies. Cucurbitacin B and E result from the acetylation of D and I, respectively, and are the most common. Cucurbitacin E and I differ, respectively, from cucurbitacin B and D by the presence of a double bond between C-1 and C-2. Cucurbitacin G and H have the same structure but differ in the configuration of the hydroxyl group at C-24. The same occurs with Cucurbitacin J and K. Cucurbitacin R was demonstrated to be 23,24‐dihydrocucurbitacin D (DHCD) being placed to the Cucurbitacin D group. Momordicosides, named after *Momordica charantia*, are a special group of Cucurbitacins which common feature is the presence of an aldehyde group at C-19 (Kaushik et al., 2015, Alghasham, 2013, Samuel, 2019, Mir et al., 2019).

**2. Physical and chemical properties**

Cucurbitacins are usually crystalline or in the form of needles, at room temperature, with exception of Cucurbitacin H which is an amorphous solid. Their chemical structure reveals they have hydrophobic properties, hence their poor water solubility but with good solubility in petroleum ether, chloroform, benzene, ethyl acetate, methanol, and ethanol. Cucurbitacins usually have absorption maxima for ultraviolet light between 228 and 234 nm. The chemically reactive positions are the ketone and alcohol groups (Alghasham, 2013, Gry et al., 2006). The molecular formula and physical properties of some Cucurbitacins are given in Table S1 (Kaushik et al., 2015).

**Table S1.** Molecular formula and physical properties of some Cucurbitacins.

| **Cucurbitacin** | **Nature** | **Formula** | **Mass** | **Melting point (ºc)** | **UV max (ethanol) nm** |
| --- | --- | --- | --- | --- | --- |
| **A** | crystals | C_32_H_46_O_9_ | 574,314 | 207-208 | 229,290 |
| **B** | crystals | C_32_H_46_O_8_ | 558,3192 | 184-186 | - |
| **C** | crystals | C_32_H_48_O_8_ | 560,3348 | 207-207,5 | 231,298 |
| **D** | crystals | C_30_H_44_O_7_ | 516,3087 | 151-153 | 230 |
| **E** | crystals | C_32_H_48_O_8_ | 556,3035 | 234,5 | 234,268 |
| **F** | crystals | C_30_H_46_O_7_ | 518,3243 | 244-245 | - |
| **G** | crystals | C_30_H_52_O_9_ | 534,3192 | 150-152 | - |
| **H** | amorphous | C_30_H_46_O_8_ | 534,3193 | - | - |
| **I** | crystals | C_30_H_42_O_7_ | 514,293 | 148-148,5 | 234,266 |
| **J** | crystals | C_30_H_44_O_8_ | 532,3036 | 200-202 | 270 |
| **K** | crystals | C_30_H_44_O_8_ | 532,3036 | 200-202 | 270 |
| **L** | crystals | C_30_H_44_O_7_ | 516,3087 | 137-142 | 270 |
| **O** | **-** | C_30_H_46_O_7_ | 518,3243 | 122-127 | - |
| **P** | **-** | C_30_H_48_O_7_ | 520,3399 | - | - |
| **Q** | **-** | C_32_H_48_O_8_ | 560,3348 | - | - |
| **S** | **-** | C_30_H_42_O_6_ | 498,298 | - | - |

**3. Synthesis and isolation**

The levels of Cucurbitacins are different according to plant tissues, being more concentrated in fruits and roots but very low in seeds (Kaushik et al., 2015). Its biosynthesis initiates with the cyclization of 2,3-oxidosqualene to cucurbitadienol by initial conversion to the protosterol cation with posterior rearrangement to the cationic lanostane system. Then, the conversion to a cucurbitane skeleton is thought to occur through a methyl shift from C-10 to C-9 followed by a hydride shift from C-5 to C-10 with the loss of a proton from C-6 (**Figure S4**) (Bucknam and
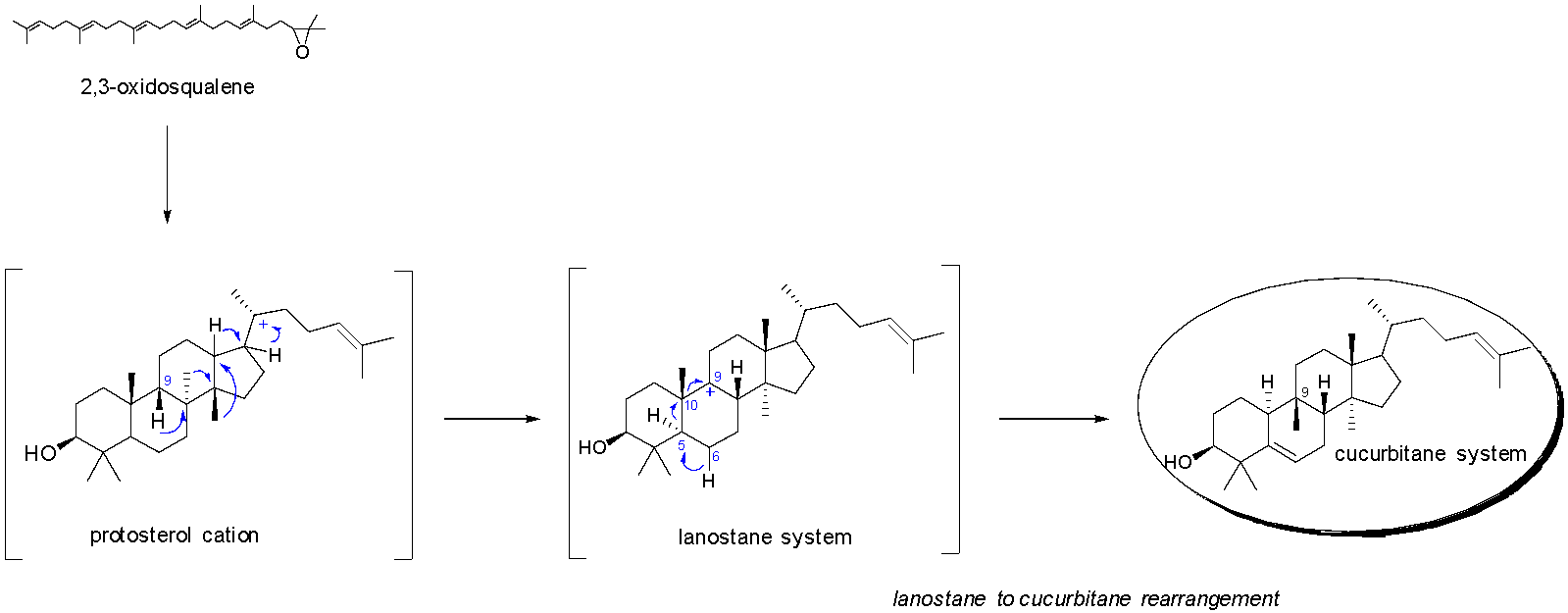
Micalizio, 2022).

**Figure S4.** Proposed conversion of 2,3-oxidosqualene to the cucurbitane system. (Adopted from (Bucknam and Micalizio, 2022)).

This transformation leads to cucurbitadienol which will then be metabolized producing several types of cucurbitacins (**Figure S5**) being considered an important intermediate in their biogenesis. For this purpose, several enzymes are involved and cucurbitadienol synthase is the first key pathway-specific enzyme. The presence of hydroxyl groups at C-2, C-3, C-19 and C-24, or a ketone function at C-3, a double bond between C-23/C-24, and an acetyl group at C-26 results in different cucurbitacins (Gry et al., 2006, Cardenas et al., 2019, Maja et al., 2022). The first cucurbitacins formed are cucurbitacin B and E. By the action of acetyl esterases, the others are produced from the primary cucurbitacins and these can be modified using a specific cucurbitacin Δ^23^-reductase (**Figure S5**) (Gry et al., 2006).


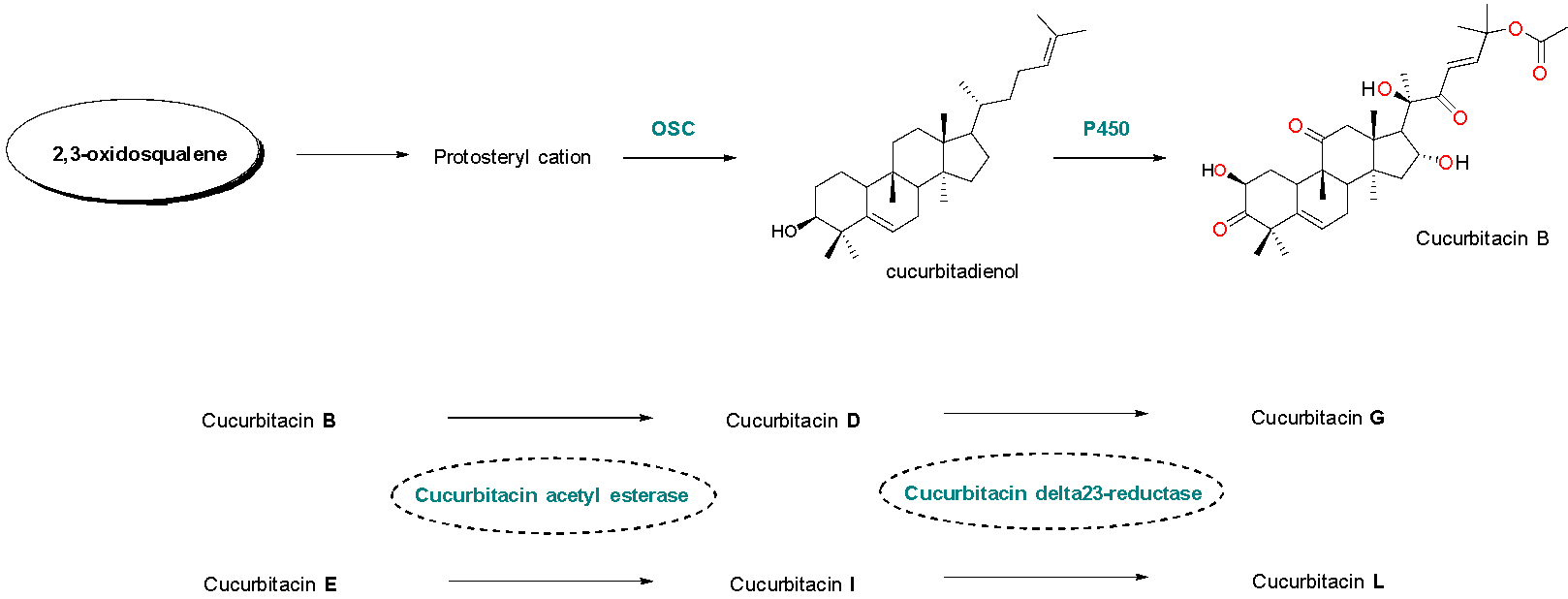


**Figure S5.** Simplified representation of the biosynthesis of triterpenoids in plants and the enzymes involved in cucurbitacins biosynthesis. OSC, oxidosqualene cyclase; P450, cytochrome P450 (Gry et al., 2006, Cardenas et al., 2019).

Cucurbitacins are secreted in scarce quantities, which has prompted to improve isolation procedures. They are moderately polar compounds and are generally extracted with methanol or ethanol. The aglycone portion has low solubility in water but is moderate in polar solvents such as chloroform. A mixture of absolute ethanol and lead acetate is also used to extract cucurbitacins. The partition between two solvents allows the purification of cucurbitacins extracted. The extraction methods most frequently used are maceration followed by fractionation via open column chromatography or thin layer chromatography. The extraction techniques selection is of capital importance since it can affect the quality and quantity of the products obtained. Recently, the extraction of bioactive compounds like cucurbitacins has explored non-conventional methods like ultrasound-assisted extraction, supercritical fluid extraction and microwave-assisted extraction (Kaushik et al., 2015, De Monte et al., 2014, Attar and Ghane, 2018).

The biological interest in cucurbitacins and the remarkable features of their core structure justifies the focus on its chemical synthesis. Jung and Lui have disclosed the construction of the tetracyclic triterpenoids cucurbitacins B and D via a multi-step strategy (**Figure S6**). They concluded that a Diels-Alder reaction was possible to construct the nucleus of cucurbitacins and that the stereochemical connection between C-8/C-9 and C-10 requires that the reaction occurs in an exoselective manner (Jung and Lui, 2010, Faisal et al., 2019).


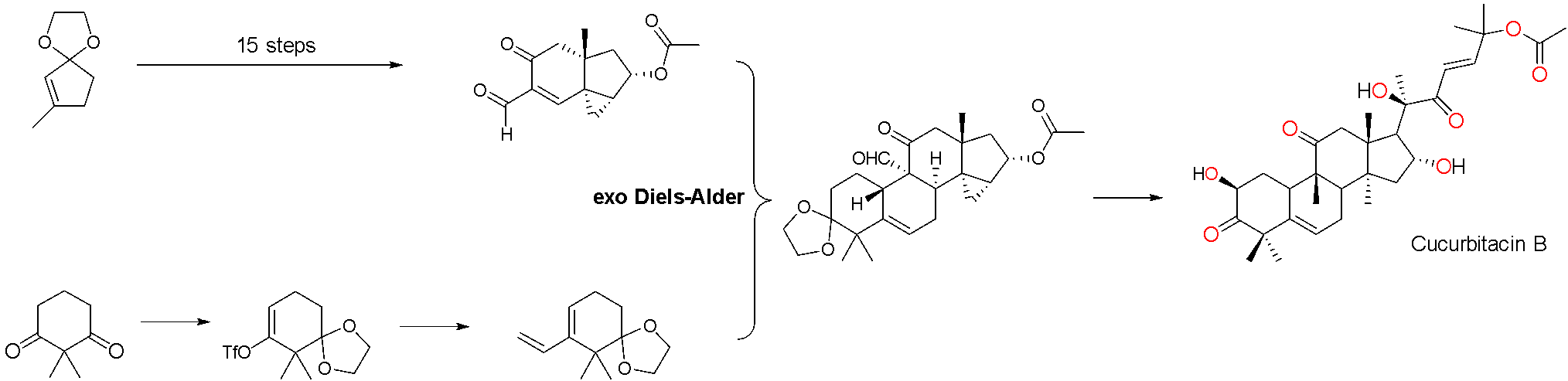


**Figure S6**. Simplified total synthesis of cucurbitacin B.

**4. Synthesis of derivatives of Cucurbitacins**

The importance of cucurbitacins as natural products in drug research and development has prompted investigation using them as templates for synthetic or semisynthetic modifications preparing derivatives with pharmacological activities. Besides that, cucurbitacins already in the study, like cucurbitacin B, present some issues namely high toxicity, low selectivity, and narrow treatment window, which make them unlikely to be drug-able agents (Chai et al., 2018). In the structure of cucurbitacins, modifications such as oxidation, reduction, acetylation, glycosylation and esterification, usually occur at C-2, C-3, C-16, C-24, and C-25 and there are many examples of cucurbitacins derivatives based on these structural changes (Mu et al., 2020). Therefore, cucurbitacin derivatives prepared based on the removal or modification of functional groups allowed to explore how those changes in the main structure, conformation, polarity, or solubility would affect the biological activity.

Some studies are focusing on the synthesis of cucurbitacin B, D, dihydrocucurbitacin B, cucurbitacin IIa derivatives, and cucurbitacin B derivatives (Cai et al., 2015, Lang et al., 2012, Guo et al., 2013). It was observed that derivatives prepared by acetylation or alkylation revealed better pharmacological activities than natural compounds (Guo et al., 2013).

The group of Lang and Silva has been synthesizing cucurbitacin derivatives for a while to afford important structure-activity relationships (SAR) studies exploring essentially anticancer properties. In 2012, they have designed cucurbitacins analogues with structural modifications in positions 2, 3, 7, 16, 20, 22 and 25 of cucurbitacin B and dihydrocucurbitacin B (**Figure S7**). Changes at C-2 and C-16 accounted for oxidation with the synthesis of disphenol derivatives; protection of the hydroxyl groups with acetic anhydride, succinic anhydride and benzoyl chloride led to diesters. Another structural change performed was based on deoxygenation reactions to study the influence of the absence of hydroxyl groups at C-2 and C-16. Hence, a modified Barton-McCombie deoxygenation reaction was used. The influence of the side chain on the biological activity was also studied. And for that purpose, a molecular simplification approach was used. Hence, it began with dibenzoyl esterification proceeded by a lithium tri-tert-butoxyaluminium hydride reduction to yield the vicinal diol derivative. This was in turn submitted to oxidative cleavage of the α-diol on the side chain with periodic acid giving the intermediate without the side chain. Still in the side chain, Lang studied at that time the importance of some key functionalities namely α,β-unsaturated ketone, the tertiary hydroxyl at C-20 and the ester at C-25, Luche reduction led to allylic alcohol, the tertiary hydroxyl at C-20 was protected as a trifluoroacetate ester, and C-25 acetate was subjected to Alvarez-Manzaneda elimination conditions yielding an alkene. Since the α, and β-unsaturated ketone in the side chain appears to have strong cytotoxicity in natural cucurbitacins, this function was introduced in rings A and B via selective oxidation using Oxone followed by elimination (Lang et al., 2012, Silva et al., 2016, Silva et al., 2015, Marostica et al., 2015).


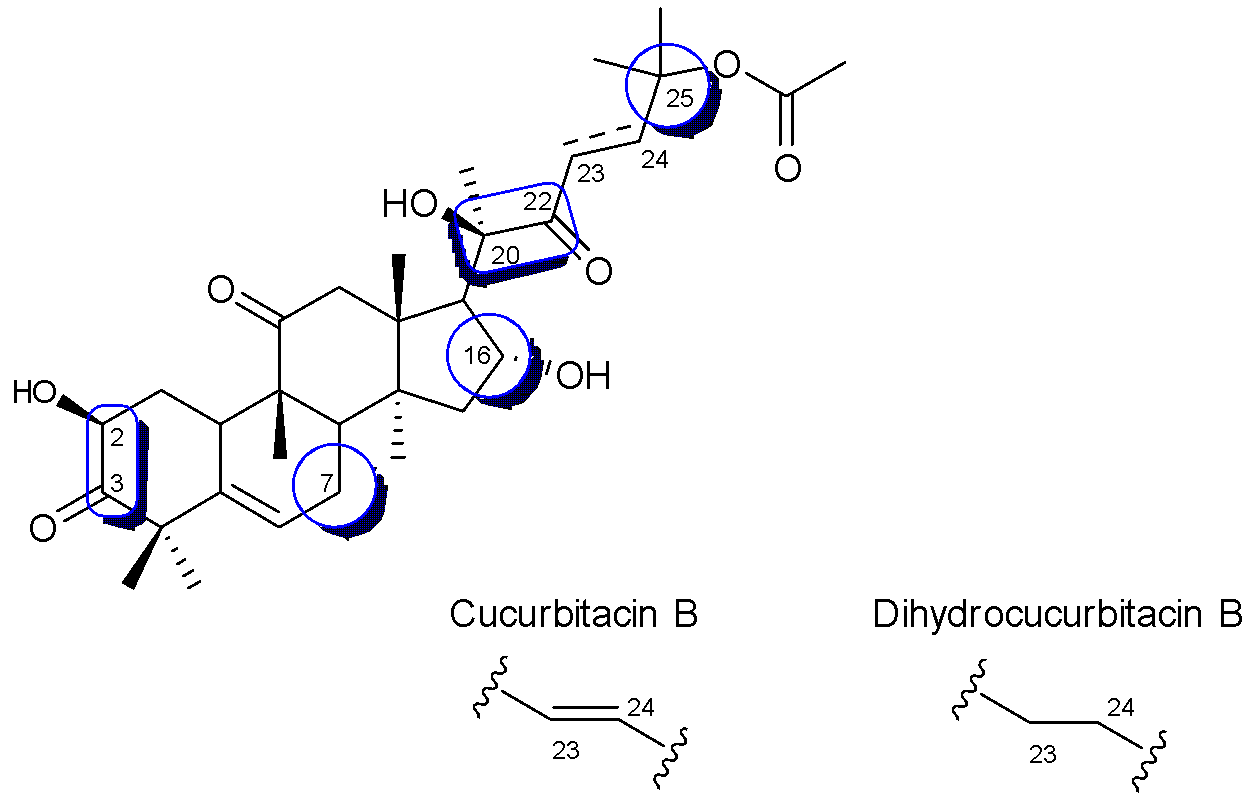


**Figure S7.** Highlighted positions (2, 3, 7, 16, 20, 22 and 25) within cucurbitacin B and dihydrocucurbitacin B that were submitted to chemical transformations.

Despite being isolated from the extract of the fruits of *E. chinensis*, elaeocarpucin C was successfully semi-synthesized to proceed for biological studies. Therefore, the strategy involved the oxidation of the allylic methyl group at C-27 of 16α,23α-epoxy-3β,20β-dihydroxy-10α,23β-cucurbit-5,24-dien-11-one using selenium dioxide as oxidizing reagent forming the desired elaeocarpucin C in sufficient yield for subsequent assays (**Figure S8**) (Pan et al., 2012).

**
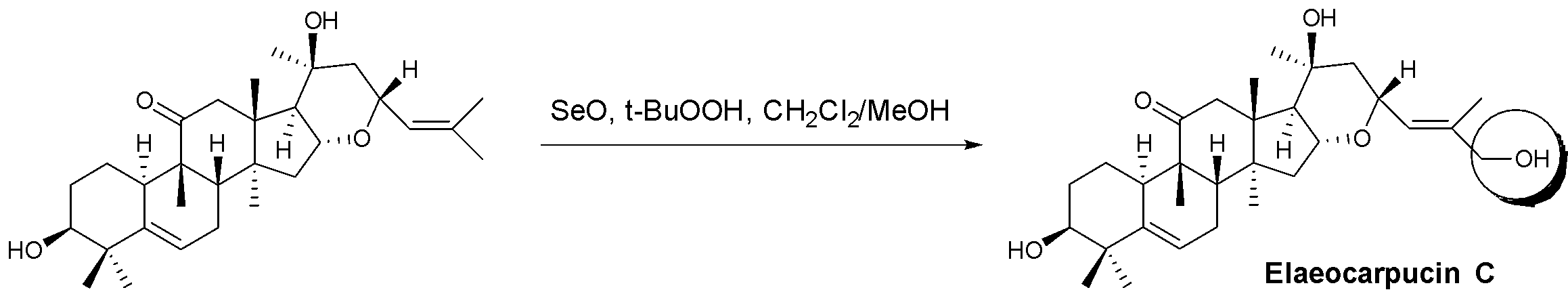
**

**Figure S8.** Semi-synthesis of elaeocarpucin C.

Three glycosylated derivatives of dihydrocucurbitacin B, 16-(1,2-orthoacetate-3,4,6-tri-*O*-acetyl-α-D-glucopyranosyl)-dihydrocucurbitacin B (**2**), 2-*O*-b- D-2,3,4,6-tetra-*O*-acetyl-galactopyranosyl dihydrocucurbitacin B (**3**) and 2-*O*-b-D-galactopyranosyl dihydrocucurbitacin B (**4**) (**Figure S9**), were synthesized for the first time via Köenigs-Knorr and imidate reaction conditions. They were then studied in a lung cancer cell line and the results contributed to QSAR (Quantitative Structure-Activity Relationships) (Lang et al., 2014, Machado et al., 2015).


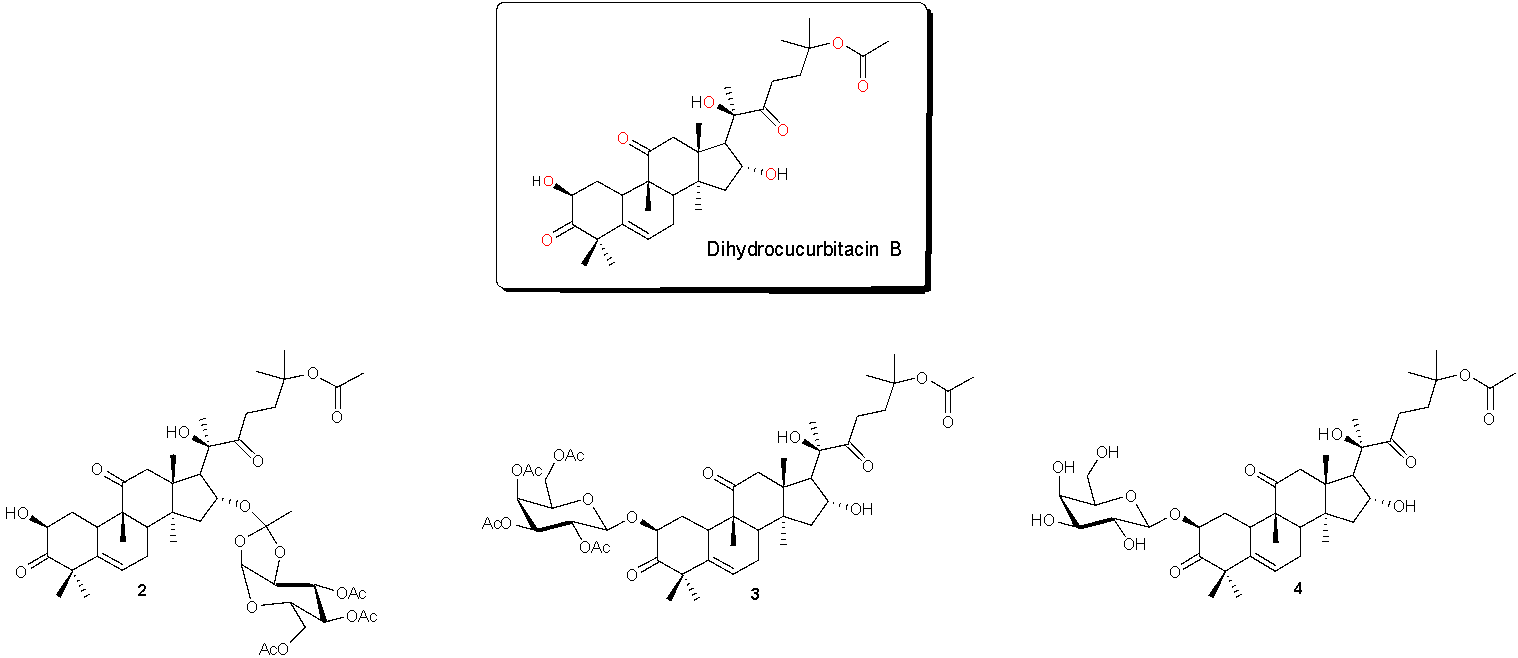


**Figure S9.** Glycosylated derivatives of dihydrocucurbitacin B.

Morotti and coworkers have synthesized eight new cucurbitacin glycosides to study the role of substituents at C-2 of dihydrocucurbitacin B and cucurbitacin B, in several biological activities. O-glycosides of dihydrocucurbitacin were synthesized by direct O-glycosylation at the C-2 position of the derivative 16-oxo-dihydrocucurbitacin B. Spacer groups between the monosaccharide moiety and the cucurbitane scaffold are usually used to join the two blocks, and in this case, 1,2,3 triazole groups were selected. Classic S_N_2 reactions allowed the synthesis of various derivatives via a chain extension at C-2 containing terminal bromine. The replacement of the bromine with azide allowed obtaining new triazole glycosylated dihydrocucurbitacin B derivatives via cycloaddition reaction with four different monosaccharides containing terminal alkynes. Deprotection of the monosaccharide units from the former derivatives was made before the cycloaddition reaction between the azide derivative and the unprotected alkynes giving the new derivatives. Therefore, new cucurbitacin glycosides, the protected triazoles and unprotected triazoles (**Figure S10**) were obtained and allowed to study substituents in the C-2 position of dihydrocucurbitacin B in several biological activities (Morotti et al., 2015).


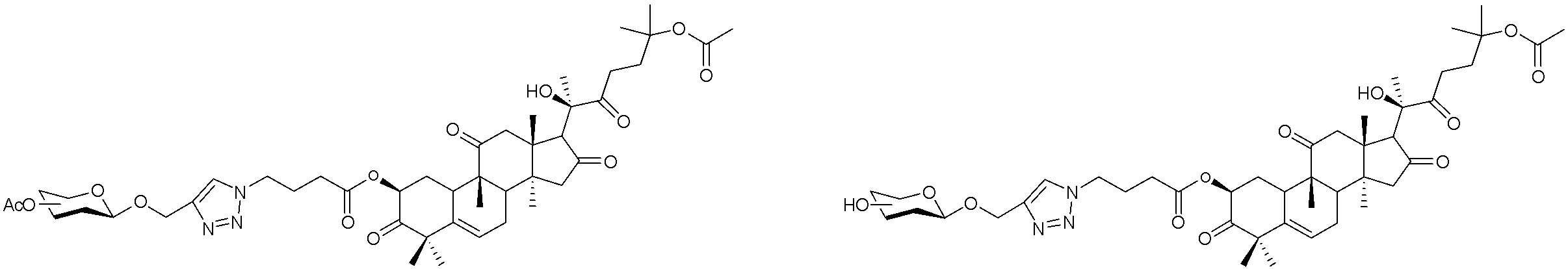


**Figure S10.** Cucurbitacins glycosides, protected triazoles (left) and unprotected triazoles (right).

Another set of cucurbitacin B derivatives was prepared using cucurbitacin B as starting substrate. Briefly, cucurbitacin B was mixed with TBSCl and imidazole in dichloromethane forming the protected derivative. To a dichloromethane solution of this, together with EDCl, DMAP and the corresponding acid, triethylamine was added forming derivatives **3a**-**3q**. Compound **4** was prepared using the same procedure as before but directly from cucurbitacin B with cinnamic acid. As for compounds **9**, **10** and **11**, they were prepared by a reaction between two other compounds prepared before with HOAc and TBAF in tetrahydrofuran. The prepared derivatives (**3a-3q**, **4**, **9a-9c**, **10a-10o**, and **11**) (**Figure S11**) were used for further SAR studies as potential anti-hepatocellular carcinoma agents revealed to have significant activity with low toxicity (Ge et al., 2018).

**
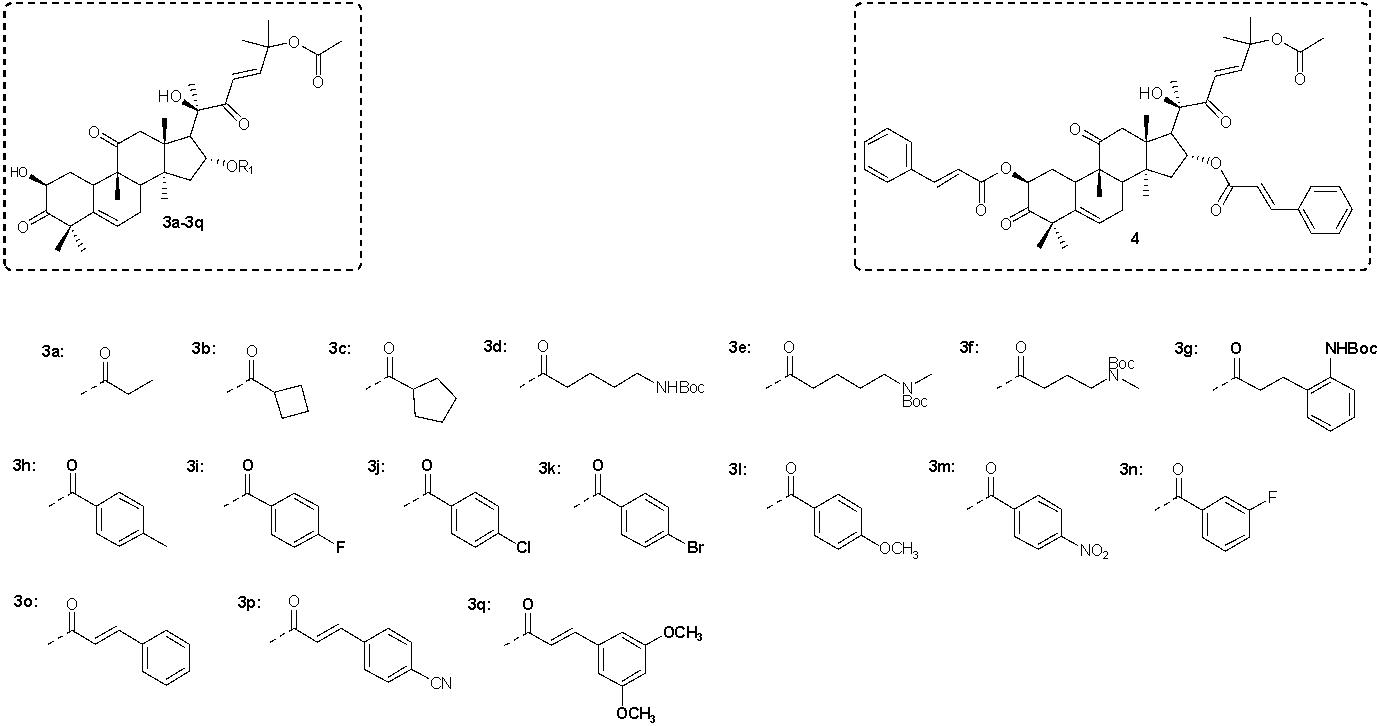
**

**
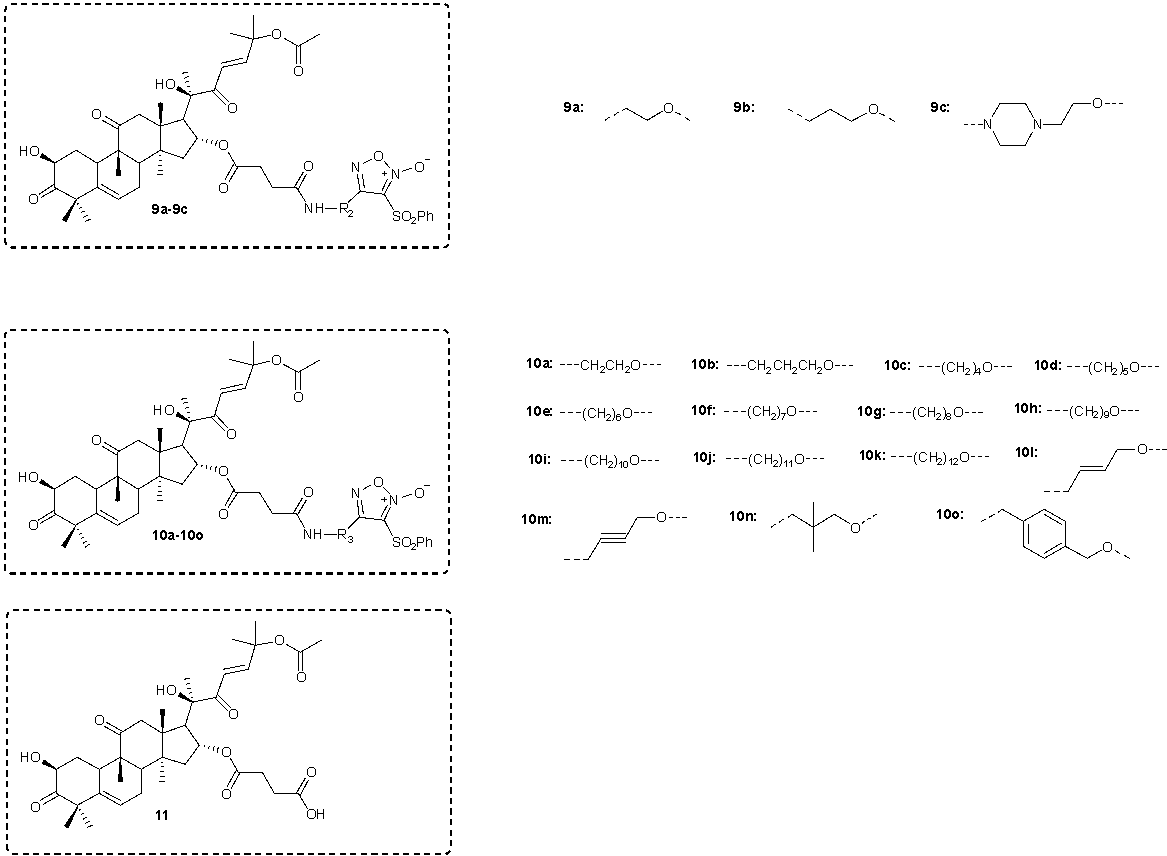
Figure S11.** Structures of cucurbitacin B derivatives prepared by (Ge et al., 2018) (Fig. A and B).

Zhang *et al.* have also synthesized cucurbitacin B and dihydrocucurbitacin B derivatives (**B1-7** and **B11** and **DB1-7** and **DB-11**, respectively) and also 3-epi-isocucurbitacin B and 3-epi-iso-dihydrocucurbitacin B derivatives (**B8-10** and **DB8-10**, respectively) (**Figure S12**) to the influence that some functional groups would have in the biological activity. Compounds **B1**, **B2** and **DB1** and **DB2** were prepared to explore the impact of hydroxyl groups at C-2 and C-16 by a Dess-Martin periodinane reaction, while esters **B3-7** and **DB3-7** were obtained by treatment of cucurbitacin B and dihydrocucurbitacin B with acetic, propionic, succinic, or benzoic anhydrides. Derivatives **B8-10** and **DB8-10** were prepared to analyze the importance of the hydroxyl group in C-3, and for that 3-epi-isocucurbitacin B and 3-epi-iso-dihydrocucurbitacin B were treated with acetic anhydride. Compounds **B11** and **DB11** were prepared to study the role of the C-7 allylic position and for that compounds **B5** and **DB3** were, respectively, oxidized with chromium trioxide and pyridine in dichloromethane (Hall et al., 2015, Zhang et al., 2020, Rosales et al., 2012).


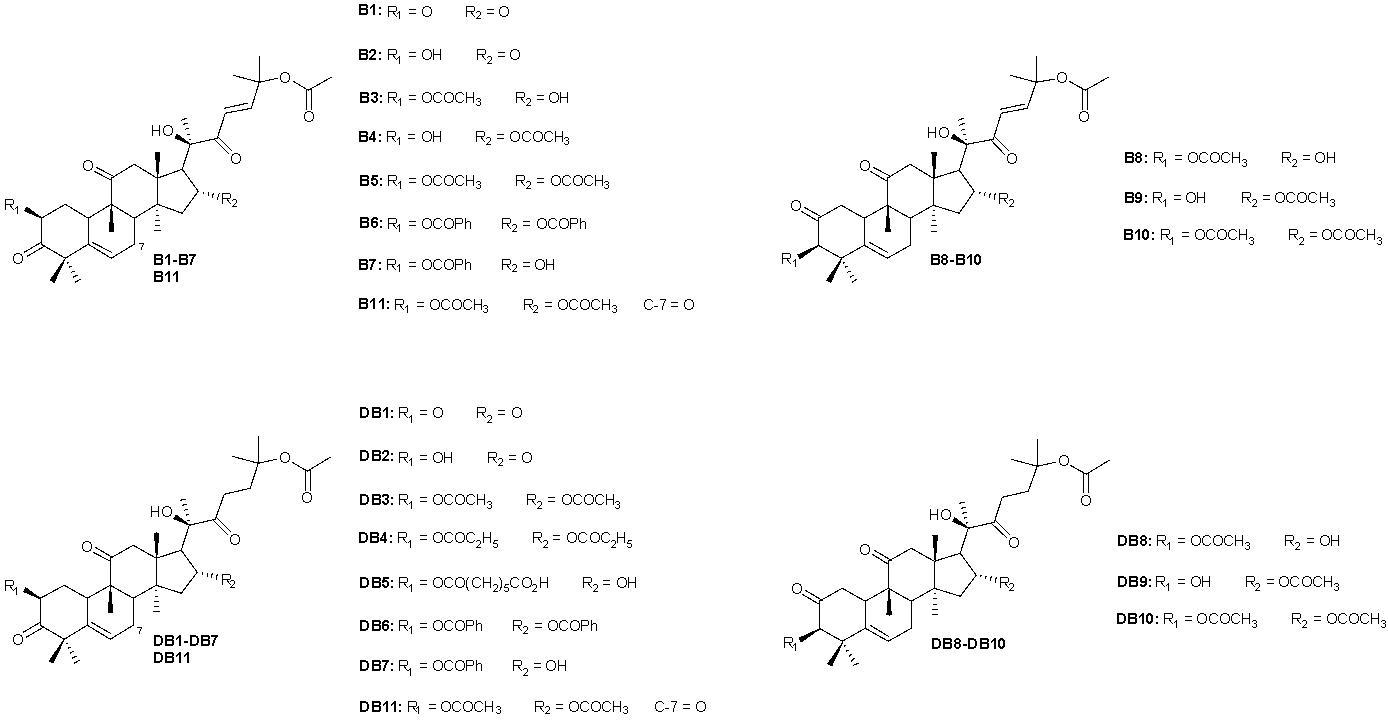


**Figure S12.** Structure of cucurbitacin B, dihydrocucurbitacin B, 3-epi-isocucurbitacin B and 3-epi-iso-dihydrocucurbitacin B derivatives prepared by Zhang *et al.*

Cucurbitacin IIA derivatives have also been prepared due to the evidence they exhibited significant potential as new drugs. In 2020, *Yu et al.*designed and synthesized twenty-one derivatives (**2**, **3**, **4a-4f**, **5a**, **5b**, **6a**, **6b**, **7**, **9a-9h**) (**Figure S13**) where the C-2, C-3, and C-16 hydroxyl groups were protected using the acetyl group, and also with different types of oxime and hydrazone branches at C-20/C-22. Briefly, cucurbitacin IIa was extracted from Hemsleya and then used as starting material reacting with DMAP and acetic anhydride in the presence of pyridine to afford compound **2**, which then reacted with sodium borohydride in methanol followed by reaction with sodium periodate in tetrahydrofuran and water forming compound **3**. Consecutive reactions of **3** with hydrazine in acetic acid gave the corresponding hydrazones **4a-4f**. As for compounds **5a** and **5b**, they were prepared mixing **3** with hydroxylamine hydrochloride in pyridine. Also, consecutive reactions of **3** with sodium hypochlorite in methanol gave the corresponding methyl ester (**7**) which was then hydrolyzed with sodium hydroxide in methanol to the corresponding carboxylic acid that was then submitted to consecutive reactions with DMTMM, N-methylmorpholine, and amine in methanol giving the corresponding amides **9a-9h** (Yu et al., 2020).


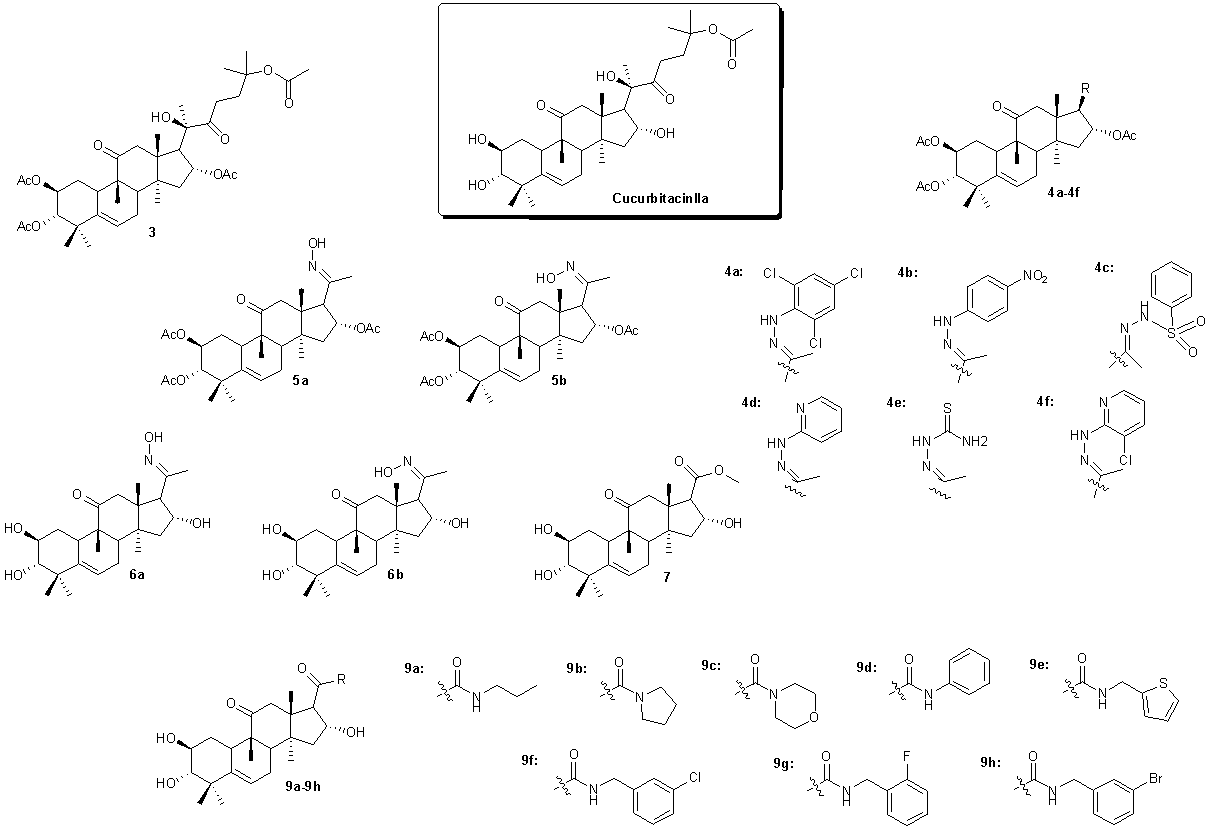
**Figure S13.** Structure of Cucurbitacin IIA derivatives prepared by *Yu* *et al.*

Octanorcucurbitacin B (**Figure S14**), a cucurbitane triterpenoid isolated from *Momordica charantia,* was recently synthesized by Bucknam and Micalizio. Its synthesis is via the direct connection of the cucurbitane skeleton without further lanostane rearrangement. This asymmetric total synthesis was achieved via a 15 step-strategy starting from a chiral enyne prepared from epichlorohydrin with the application of a sequence of stereoselective metallacycle-mediated annulative cross-coupling followed by a stereoselective intramolecular Heck reaction forming the “steroidal” C-9/C-10 bond as an enantioselective entry to the overall cucurbitane framework (Bucknam and Micalizio, 2022).


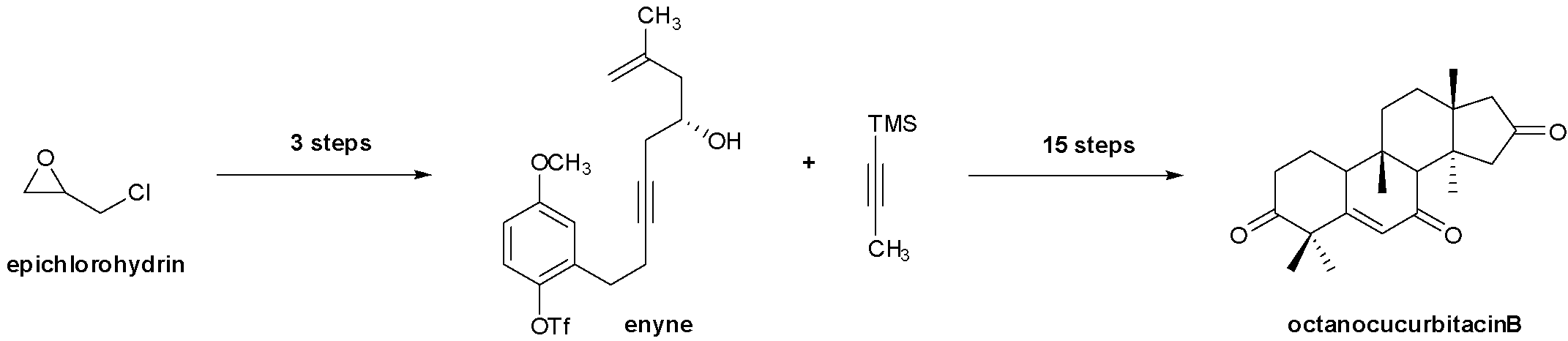
**Figure S14.** The simplified overall scheme of synthesis of octanorcucurbitacin B.

**References**

ALGHASHAM, A. A. 2013. Cucurbitacins - a promising target for cancer therapy. *Int J Health Sci (Qassim),* 7**,** 77-89.

ATTAR, U. & GHANE, S. 2018. Optimized extraction of anti-cancer compound–cucurbitacin I and LC–MS identification of major metabolites from wild Bottle gourd (Lagenaria siceraria (Molina) Standl.). *South African Journal of Botany,* 119**,** 181-187.

BUCKNAM, A. R. & MICALIZIO, G. C. 2022. Asymmetric De Novo Synthesis of a Cucurbitane Triterpenoid: Total Synthesis of Octanorcucurbitacin B. *J Am Chem Soc,* 144**,** 8493-8497.

CAI, Y., FANG, X., HE, C., LI, P., XIAO, F., WANG, Y. & CHEN, M. 2015. Cucurbitacins: A Systematic Review of the Phytochemistry and Anticancer Activity. *Am J Chin Med,* 43**,** 1331-50.

CARDENAS, P. D., ALMEIDA, A. & BAK, S. 2019. Evolution of Structural Diversity of Triterpenoids. *Front Plant Sci,* 10**,** 1523.

CHAI, Y., XIANG, K., WU, Y., ZHANG, T., LIU, Y., LIU, X., ZHEN, W. & SI, Y. 2018. Cucurbitacin B Inhibits the Hippo-YAP Signaling Pathway and Exerts Anticancer Activity in Colorectal Cancer Cells. *Med Sci Monit,* 24**,** 9251-9258.

CHEN, X., BAO, J., GUO, J., DING, Q., LU, J., HUANG, M. & WANG, Y. 2012. Biological activities and potential molecular targets of cucurbitacins: a focus on cancer. *Anticancer Drugs,* 23**,** 777-87.

DE MONTE, C., CARRADORI, S., GRANESE, A., DI PIERRO, G. B., LEONARDO, C. & DE NUNZIO, C. 2014. Modern extraction techniques and their impact on the pharmacological profile of Serenoa repens extracts for the treatment of lower urinary tract symptoms. *BMC urology,* 14**,** 1-11.

FAISAL, M., SAEED, A. & SHAHZAD, D. 2019. Portrait of the synthesis of some potent anti-inflammatory natural products. *Discovery and Development of Anti-Inflammatory Agents from Natural Products.*

GE, W., CHEN, X., HAN, F., LIU, Z., WANG, T., WANG, M., CHEN, Y., DING, Y. & ZHANG, Q. 2018. Synthesis of cucurbitacin B derivatives as potential anti-hepatocellular carcinoma agents. *Molecules,* 23**,** 3345.

GRY, J., SØBORG, I. & ANDERSSON, H. 2006. Identity physical and chemical properties and analytical methods. *Cucurbitacins in plant food. Tema Nord Nordic Council of Ministers. Print: Ekspressen Tryk & Kopicenter. Copenhagen,* 556**,** 17-22.

GUO, R. H., GENG, C. A., HUANG, X. Y., MA, Y. B., ZHANG, Q., WANG, L. J., ZHANG, X. M., ZHANG, R. P. & CHEN, J. J. 2013. Synthesis of hemslecin A derivatives: a new class of hepatitis B virus inhibitors. *Bioorg Med Chem Lett,* 23**,** 1201-5.

HALL, J. A., SEEDARALA, S., RICE, N., KOPEL, L., HALAWEISH, F. & BLAGG, B. S. 2015. Cucurbitacin D is a disruptor of the HSP90 chaperone machinery. *Journal of natural products,* 78**,** 873-879.

JUNG, M. E. & LUI, R. M. 2010. Studies toward the total syntheses of cucurbitacins B and D. *J Org Chem,* 75**,** 7146-58.

KAUSHIK, U., AERI, V. & MIR, S. R. 2015. Cucurbitacins - An insight into medicinal leads from nature. *Pharmacogn Rev,* 9**,** 12-8.

LANG, K. L., SILVA, I. T., MACHADO, V. R., ZIMMERMANN, L. A., CARO, M. S., SIMÕES, C. M., SCHENKEL, E. P., DURÁN, F. J., BERNARDES, L. S. & DE MELO, E. B. 2014. Multivariate SAR and QSAR of cucurbitacin derivatives as cytotoxic compounds in a human lung adenocarcinoma cell line. *Journal of Molecular Graphics and Modelling,* 48**,** 70-79.

LANG, K. L., SILVA, I. T., ZIMMERMANN, L. A., MACHADO, V. R., TEIXEIRA, M. R., LAPUH, M. I., GALETTI, M. A., PALERMO, J. A., CABRERA, G. M., BERNARDES, L. S., SIMÕES, C. M., SCHENKEL, E. P., CARO, M. S. & DURÁN, F. J. 2012. Synthesis and cytotoxic activity evaluation of dihydrocucurbitacin B and cucurbitacin B derivatives. *Bioorg Med Chem,* 20**,** 3016-30.

MACHADO, V. R., LANG, K. L., DURÁN, F. J., CABRERA, G. M., PALERMO, J. A., SCHENKEL, E. P. & BERNARDES, L. S. 2015. Di-hidrocucurbitacina B: Semi-síntese de novos derivados glicosilados. *Química Nova,* 38**,** 37-41.

MAJA, D., MAVENGAHAMA, S. & MASHILO, J. 2022. Cucurbitacin biosynthesis in cucurbit crops, their pharmaceutical value and agricultural application for management of biotic and abiotic stress: A review. *South African Journal of Botany,* 145**,** 3-12.

MAROSTICA, L. L., SILVA, I. T., KRATZ, J. M., PERSICH, L., GELLER, F. C., LANG, K. L., CARO, M. S., DURAN, F. J., SCHENKEL, E. P. & SIMOES, C. M. 2015. Synergistic Antiproliferative Effects of a New Cucurbitacin B Derivative and Chemotherapy Drugs on Lung Cancer Cell Line A549. *Chem Res Toxicol,* 28**,** 1949-60.

MIR, S. A., MUKHERJEE, S., MAKAR, S. & PAL, G. 2019. Cucurbitacins a vibrant triterpenoid: a review on its anticancer property. *PharmaTutor,* 7**,** 43-54.

MOROTTI, A. L., LANG, K. L., CARVALHO, I., SCHENKEL, E. P. & BERNARDES, L. S. 2015. Semi-synthesis of new glycosidic triazole derivatives of dihydrocucurbitacin B. *Tetrahedron Letters,* 56**,** 303-307.

MU, S., LI, J., LIU, C., ZENG, Y., MEN, Y., CAI, Y., CHEN, N., MA, H. & SUN, Y. 2020. Effective glycosylation of cucurbitacin mediated by UDP-glycosyltransferase UGT74AC1 and molecular dynamics exploration of its substrate binding conformations. *Catalysts,* 10**,** 1466.

PAN, L., YONG, Y., DENG, Y., LANTVIT, D. D., NINH, T. N., CHAI, H., CARCACHE DE BLANCO, E. J., SOEJARTO, D. D., SWANSON, S. M. & KINGHORN, A. D. 2012. Isolation, structure elucidation, and biological evaluation of 16,23-epoxycucurbitacin constituents from Eleaocarpus chinensis. *J Nat Prod,* 75**,** 444-52.

ROSALES, A., MUÑOZ-BASCÓN, J., MORALES-ALCÁZAR, V. M., CASTILLA-ALCALÁ, J. A. & OLTRA, J. E. 2012. Ti (III)-catalyzed, concise synthesis of marine furanospongian diterpenes. *RSC advances,* 2**,** 12922-12925.

SAMUEL, A. 2019. Cucurbitacins and its Anticancer property: A Review. *Himalayan Journal of Health Sciences***,** 17-23.

SILVA, I. T., CARVALHO, A., LANG, K. L., DUDEK, S. E., MASEMANN, D., DURAN, F. J., CARO, M. S., RAPP, U. R., WIXLER, V., SCHENKEL, E. P., SIMOES, C. M. & LUDWIG, S. 2015. In vitro and in vivo antitumor activity of a novel semisynthetic derivative of cucurbitacin B. *PLoS One,* 10**,** e0117794.

SILVA, I. T., GELLER, F. C., PERSICH, L., DUDEK, S. E., LANG, K. L., CARO, M. S., DURÁN, F. J., SCHENKEL, E. P., LUDWIG, S. & SIMÕES, C. M. 2016. Cytotoxic effects of natural and semisynthetic cucurbitacins on lung cancer cell line A549. *Invest New Drugs,* 34**,** 139-48.

YU, K., YANG, X., LI, Y., CUI, X., LIU, B. & YAO, Q. 2020. Synthesis of cucurbitacin IIa derivatives with apoptosis-inducing capabilities in human cancer cells. *RSC Adv,* 10**,** 3872-3881.

ZHANG, X., LI, H., WANG, W., CHEN, T. & XUAN, L. 2020. Lipid-lowering activities of cucurbitacins isolated from Trichosanthes cucumeroides and their synthetic derivatives. *Journal of Natural Products,* 83**,** 3536-3544.
